# Pro‐ and Anti‐Inflammatory Macrophages Adjust UCP2 Protein Levels Based on Their Intrinsic Metabolism and Available Metabolites

**DOI:** 10.1002/eji.70218

**Published:** 2026-06-09

**Authors:** Jila Nasirzade, Felix Sternberg, Andrea Vogel, Roko Sango, Taraneh Beikbaghban, Thomas Kolbe, Thomas Rattei, Thomas Weichhart, Elena E. Pohl

**Affiliations:** ^1^ Physiology and Biophysics Department of Biological Science and Pathobiology University of Veterinary Medicine Vienna Austria; ^2^ Department of Nutritional Sciences Faculty of Life Sciences University of Vienna Vienna Austria; ^3^ Center of Pathobiochemistry and Genetics Institute of Medical Genetics Medical University of Vienna Vienna Austria; ^4^ Centre for Microbiology and Environmental Systems Science University of Vienna Vienna Austria; ^5^ Doctoral School in Microbiology and Environmental Science University of Vienna Vienna Austria; ^6^ Laboratory Animal Medicine Department of Biological Science and Pathobiology University of Veterinary Medicine Vienna Austria; ^7^ Department of Agricultural Sciences University of Natural Resources and Life Sciences Vienna Austria

**Keywords:** cobalt chloride, extracellular acidification rate, glycolysis, oxygen consumption rate, RAW 264.7 cells, SLC25A8, tissue‐resident macrophages, UK5099

## Abstract

The immune and metabolic responses of macrophages are closely linked. Mitochondrial uncoupling protein 2 (UCP2), proposed to facilitate metabolite transport, is involved in regulating inflammation and glucose metabolism in macrophages. However, its significance and regulatory mechanism in subsets of macrophages with distinct metabolic profiles remain unclear. In this study, we demonstrate that under physiological nutrient conditions, inflammatory stimuli in classically activated macrophages (via LPS) reduce UCP2 expression in line with decreased oxygen consumption rates, indicating mitochondrial suppression. In contrast, alternatively activated macrophages (via IL4) displayed higher UCP2 levels and enhanced respiration. Under glucose deprivation, LPS‐stimulated macrophages retained mitochondrial activity despite lower UCP2 levels. Blocking pyruvate entry into the mitochondria reduced UCP2 expression, highlighting the connection between glycolysis and mitochondrial metabolism. Mimicking the hypoxic milieu characteristic of LPS‐activated macrophages through CoCl_2_ treatment of IL‐4‐activated macrophages resulted in decreased UCP2 expression, suggesting that hypoxia broadly mediates UCP2 suppression in macrophages. Overall, our findings suggest that UCP2 protein levels are modulated by metabolic alterations in macrophages, with pyruvate acting as a key regulator of UCP2 abundance. This emphasizes the importance of UCP2 in linking glycolysis with mitochondrial metabolism, providing insights for developing therapeutic strategies for diseases involving immunometabolic dysregulation.

## Graphical abstract

LPS‐stimulated macrophages exhibit high glycolytic flux, with pyruvate preferentially converted to lactate even under normoglycemic conditions, limiting pyruvate availability for the tricarboxylic acid (TCA) cycle and reducing oxygen consumption rate (OCR). Glucose deprivation increases OCR; however, an innate hypoxic state is associated with low UCP2 levels in this phenotype.

In contrast, under normal glucose conditions, IL4‐stimulated macrophages enhance mitochondrial pyruvate uptake, promoting TCA cycle activity and higher OCR. Elevated glutamine levels increase UCP2 expression, whereas hypoxia and high glucose suppress it.

Overall, UCP2 levels are dynamically regulated by pyruvate availability, supporting its role in C4 metabolite transport and enabling metabolic flexibility in response to nutrient availability.

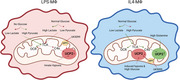

**Abbreviations**
(p)STAT: (phosphorylated) signal transducer and activator of transcription
BMDMΦ: bone marrow‐derived macrophages
ECAR: extracellular acidification rate
IFNγ: interferon γ
IL‐13: interleukin‐13
IL‐4: interleukin‐4
LPS: lipopolysaccharide
MΦ: macrophage
OCR: oxygen consumption rate
OxPhos: oxidative phosphorylation
SDHA: succinate dehydrogenase complex flavoprotein subunit a
TCA: tricarboxylic acid
TRM: tissue‐resident macrophages
UCP2: uncoupling protein 2

## 1 Introduction

Immune responses and metabolic regulation are intricately linked, both essential for the proper functioning of the organism. Disruptions in these processes can lead to a range of immunometabolism‐related diseases, including cancer, obesity, and diabetes. Immune cells adapt to dietary changes by modifying lipid and/or glucose metabolism, which, in turn, affects the intrinsic metabolism of surrounding cells crucial for maintaining metabolic homeostasis [1, 2].

The immune and metabolic responses in macrophages are tightly regulated by mitochondria. In vitro, lipopolysaccharide (LPS) and interferon γ (IFNγ) are used to polarize macrophages (MΦs) to a pro‐inflammatory phenotype (LPS‐MΦs), resulting in a shift from glucose‐dependent oxidative phosphorylation (OxPhos) to aerobic glycolysis, a process known as the Warburg effect [3]. This phenotype has an impaired tricarboxylic acid (TCA) cycle [4], making it more dependent on ATP produced by glycolysis rather than OxPhos in mitochondria [5]. On the other hand, anti‐inflammatory macrophages polarized in vitro by interleukin‐4 and ‐13 (IL4 and IL13) stimulation (IL4‐MΦs) are highly dependent on OxPhos and use glutamine as a major source of their metabolism [4]. Macrophages can rapidly switch from a catabolic to anabolic state to support the immune cell phenotype, and mitochondria help in their polarization and adaptation to environmental changes [6]. Although distinct metabolic profiles among macrophage subtypes are increasingly recognized, the mechanisms governing their selective metabolite utilization remain poorly understood.

Uncoupling protein 2 (UCP2) is a member of the large superfamily of mitochondrial anion carriers (SLC25) and is localized in the inner membrane of mitochondria. Although UCP2 was first discovered in the late 1990s [7], its biological function remains a subject of ongoing debate. UCP2 has been shown to be abundant in cells that primarily rely on glycolysis, have high proliferation rates, and possess stemness characteristics [8, 9], such as cancer cells [10, 11, 12], stem cells [13, 14, 15], and immune cells, including macrophages, monocytes, B‐cells, T‐cells, and microglia [16, 17]. Repeated stimulation has been shown to increase UCP2 abundance in T‐cells [15, 17].

UCP2 knockout experiments indicated the role of UCP2 in immune response, as UCP2 knockout mice were observed to exhibit increased resistance to *Toxoplasma gondii* infection [18]. These knockout mice also showed decreased levels of inflammatory cytokines such as IL1β and IL6 [19, 20, 21, 22]. Further evidence supporting the role of UCP2 in macrophage immune responses comes from studies showing an increase in UCP2 expression following LPS injection [23, 24]. A recent study has shown that microglia‐specific UCP2 knockout mice exhibit significant mitochondrial hyperfusion following optical nerve crush [16]. This effect was linked to an increased reliance on OxPhos for ATP production in male mice under injury‐induced conditions. In alveolar macrophages, UCP2 has been reported to limit a pathogen‐killing (pro‐inflammatory) phenotype while promoting a preresolving (anti‐inflammatory) phenotype by inducing efferocytosis of pathogen debris [25]. These studies suggest that UCP2 may provide metabolic flexibility, allowing cells to select the most appropriate metabolic substrate in response to different metabolic challenges and polarization states.

In light of these observations, the variation in metabolic pathways between LPS‐MΦ and IL4‐MΦs suggests that UCP2 levels may be regulated during macrophage polarization. Macrophages are well adapted to recognizing microenvironmental cues and are highly plastic in modifying their properties and functions accordingly [26]. The decision between maintaining glycolysis or continuing with oxidative phosphorylation makes UCP2 highly relevant to macrophage plasticity. Since UCP2 has been shown to play a role in transporting C4 metabolites [27], depending on nutrient availability, to support the flow of the TCA cycle [28], one could strongly propose its relevance in enabling macrophages to select specific metabolic pathways based on available metabolites.

In this study, we aimed to evaluate whether changes in environmental parameters influence adaptive shifts in UCP2 levels and to determine if these changes correlate with the distinct metabolic profiles of pro‐ and anti‐inflammatory macrophages under both physiological and pathological conditions. For this, we differentiated bone marrow‐derived macrophages into pro‐inflammatory and anti‐inflammatory subsets and exposed them to various metabolic challenges, such as shortage or deprivation of glucose, glutamine, and pyruvate, as well as hypoxia‐mimicking conditions. Finally, we investigated the interrelationship between UCP2 protein levels and key cellular parameters such as oxygen consumption rate (OCR), extracellular acidification rate (ECAR), cell proliferation, and oxygenation.

## 2 Materials and Methods

### 2.1 Experimental Mice

Eight‐ to ten‐week‐old female and male C57BL/6J mice were purchased from Janvier Labs (Le Genest‐Saint‐Isle, France). Upon arrival, the mice were acclimated for 4 days in a controlled environment in the animal facility at the Institute of In Vivo and In Vitro Models at the University of Veterinary Medicine Vienna. The photoperiod was 12L:12D, the temperature was maintained at 21 ± 1°C, and the humidity was maintained at 50 ± 10%. Health screening was performed according to the 2014 FELASA recommendations and confirmed the specified pathogen‐free (SPF) status of the mice. The mice were housed in polysulfone IVC cages (type IIL, Blue Line Classic, Tecniplast, Hohenpeissenberg, Germany), which were equipped with wood bedding (Lignocel Select, Rettenmaier Austria GmbH & Co. KG, Vienna, Austria) and nesting material (Pur‐Zellin, Paul Hartmann GesmbH, Wiener Neudorf, Austria). A maintenance diet for rodents (V1534, irradiated; Ssniff Spezialdiäten GmbH, Soest, Germany) and chlorinated tap water (4 ppm) were provided ad libitum. Qualified personnel performed daily checks to monitor animal health. The mice were sacrificed by cervical dislocation in accordance with the ethical approval of the internal Ethics and Animal Welfare Committee (approval number ETK‐172/11/2023).

### 2.2 Isolation of Tissue‐Resident Macrophages (TRM)

Mice were euthanized by cervical dislocation, and tissues (spleen, lung, liver, bone marrow, brain, colon, adipose tissue, and peritoneum) were rapidly collected. The tissues were minced into small pieces and digested using the Multi Tissue Digestion Kit (130‐110‐203, Miltenyi Company, California, USA) for 20 min. After digestion, single‐cell suspensions were filtered through a 70 µM strainer and centrifuged at 500 g for 3–5 min. The pellet was then resuspended in EasySep buffer (20144, StemCell, Cologne, Germany) and passed through a 40 µm strainer into 5 mL tubes. Rat serum was added to the cell suspensions, followed by incubation with the first antibody (APC or PE conjugated; #100‐0033, EasySep Release Mouse APC/PE Positive Selection Kit, StemCell, Cologne) for 5 min at room temperature. Magnetic beads were then added, and the mixture was incubated for 3 min at room temperature. The cells were topped up with EasySep buffer and placed into the EasySep Magnet (#18103, StemCell, Cologne) for 3 min. After incubation, the supernatant was carefully aspirated without touching the cells adhering to the side facing the magnet. The tube was then removed from the magnet, and cells were washed with Easy Sep buffer before being returned to the magnet for another 3 min incubation. This wash step was repeated twice. After the final wash, cells were resuspended in EasySep buffer, and release buffer concentrate was added, followed by a 3 min incubation. Subsequently, tubes were placed back in the magnet, and the supernatant containing the positively selected cells was collected. The cells were centrifuged, resuspended in EasySep buffer, and the entire procedure was repeated with a second antibody (PE or APC conjugated, using a different fluorophore than in the first step). The antibodies/markers used for the two‐step magnetic enrichment of the respective TRM population are shown in Table S1.

The specific TRM markers used for the bead‐based magnetic enrichment [29, 30] were previously verified by a flow gating strategy based on common and TRM‐specific markers. The most selective markers were chosen for the bead enrichment strategy. This enrichment process resulted in a macrophage population with >95% purity. As an example, the gating strategy and enrichment efficiency for splenic macrophages are shown in Figure S8. After the second enrichment step, cells were resuspended in Triazole, and total RNA was isolated by using Monarch RNA Cleanup Kit (T2040, New England Biolab Company, Massachusetts, USA) according to the manufacturer's instructions.

### 2.3 Isolation and Culture of Murine Bone Marrow‐Derived Macrophages (BMDMΦs) and RAW 264.7 Cells

Eight‐ to ten‐week‐old female C57Bl/6j mice were purchased from Janvier Labs (Le Genest‐St‐Isle, France). Mice were sacrificed by cervical dislocation in accordance with the ethical approval of the Austrian national authority under the Animal Experiments Act (Tierversuchsgesetz 2012; approval number ETK‐172/11/2023). The femora, tibiae, and humeri were then harvested, and bone marrow was collected by rinsing the bones with media using a syringe.

Cells were seeded at Nunc EasYDish Dishes (150468, Thermo Fisher Scientific Inc., Massachusetts, USA) and cultured under standard conditions (37°C, 5% CO_2_, and 95% humidity) for 5 days in RPMI 1640 medium (2522621, Gibco, Dublin, Ireland), supplemented with heat‐inactivated fetal bovine serum (HI FBS; 10082147, Gibco, Dublin, Ireland), 1% penicillin/streptomycin (P/S) (15140122, Gibco), and with 25 ng/mL macrophage colony‐stimulating factor (M‐CSF; 315‐02‐50UG, Peprotech, Gibco). Media change and re‐addition of macrophage colony‐stimulating factor (MCSF) was done at day 3 of culture.

On day 5, bone marrow‐derived macrophages (BMDMΦs) were harvested by scraping, counted, and reseeded into appropriate cell plates for overnight culturing with the same media. For immunoblotting and quantitative PCR analysis, 1.5 × 10^6^ cells and for the proliferation assay, 25 × 10^5^ cells were seeded per well in six‐well plates (174901, Thermo Fisher Scientific Inc.). For Seahorse extracellular analysis, 80 × 10^4^ cells were seeded per well in Seahorse 96XFe plates (102959‐100, Agilent, California, USA).

After overnight culturing, macrophages were exposed to DMEM media without glucose, glutamine, or phenol red (A1443001, Gibco), but supplemented with 10% HI FBS, 1% P/S, and 15 ng/mL M‐CSF. Unless otherwise specified, the media also contained 2 mM GlutaMAX (35050061, Gibco), 5.5 mM glucose (A2494001, Gibco), and 1 mM sodium pyruvate (11360070, Gibco).

To induce an inflammatory phenotype in BMDMΦs, 50 ng/mL of lipopolysaccharide (LPS; L8643, Sigma‐Aldrich, Massachusetts, USA) and 5 ng/mL of interferon‐γ (IFN‐γ; 315‐05, Peprotech, Gibco) were added to the media. To polarize BMDMΦs into an anti‐inflammatory phenotype, 20 ng/mL of interleukin‐4 (IL4; 214‐14, Peprotech, Gibco) and 20 ng/mL of interleukin‐13 (IL13; 200–13, Peprotech, Gibco) were added. If required for the experiment, 25 mM of lactate (1614308, Sigma Aldrich, Massachusetts, USA), 10 µM MG132 (M8699, Sigma‐Aldrich), and 10 or 50 µM of UK5099 (5.04817, Sigma Aldrich) were added.

RAW 264.7 macrophage‐like cells, kindly provided by Dr. Reinhard Gruber (Oral Biology group at the Dentistry school of Vienna), were expanded in RPMI 1640 medium supplemented with 10% FBS, 1% P/S for 4 passages. Cells were then seeded at 1 × 10^6^ cells/well for immunoblotting and 25 × 10^5^ cells/well for proliferation assays in six‐well plates. Polarization of RAW 264.7 cells was performed following the same protocol used for BMDMΦs.

### 2.4 Protein Isolation and Quantitative Immunoblot Analysis

Protein isolation from cells and immunoblot analysis were performed as described in Rupprecht et al. [15]. In brief, cells were washed with phosphate‐buffered saline (PBS) solution, collected in RIPA buffer containing a 1:50 protease inhibitor cocktail, and sonicated. Total cellular protein was isolated after centrifugation and quantified using a BCA kit (A55860, Thermo Fisher Scientific Inc.). We loaded 20 µg of total cellular protein per lane for immunoblot analyses. We used the following primary antibodies raised against: succinate dehydrogenase complex flavoprotein subunit A (anti‐SDHA, ab14715, Cambridge, UK), 2‐oxoglutarate dehydrogenase (anti‐OGDH, 15212‐1‐AP, Proteintech Group Inc, UK), voltage‐dependent anion channel (anti‐VDAC, ab14734, Abcam Inc., UK), β‐actin (A5441, Sigma Aldrich Inc.), and a self‐designed, validated antibody against UCP2 [15, 17]. Antibodies against signal transducer and activator of transcription 1 (anti‐STAT1, 9172, Cell Signaling Technology, Inc., Massachusetts, USA), p‐STAT1 (9167, Cell Signaling Technology, Inc.), signal transducer and activator of transcription 6 (anti‐STAT6, 9162, Cell Signaling Technology, Inc.), p‐STAT6 (9361, Cell Signaling Technology, Inc.), B‐cell lymphoma 2 (anti‐bcl2, 3498, Cell Signaling Technology, Inc.), Bcl‐2‐associated X protein (anti‐BAX, 2772, Cell Signaling Technology, Inc.), NFKBIA/IkB alpha (H‐4) (sc‐1643, Santa Cruz Biotechnology Inc., USA), and α‐tubulin (801202, Bio Legend Inc., San Diego, USA) were used at dilutions 1:1000 and 1:10,000, respectively.

Immunoreactions were detected by luminescence using a secondary antibody against rabbit (7074, Cell Signaling Technology, Inc.) or mouse (NA931V, GE HealthCare Technologies, Inc., Chicago, USA). Antibodies were linked to the horseradish peroxidase and ECL Immunoblot Blotting reagent (1705061, Bio‐Rad Laboratories Ges.m.b.H., California, USA). The intensity of bands was quantified using Software VisionWorks 8.20 (Analytik Jena GmbH+Co., Jena, Germany). For all Western blot analyses in this study, band intensity normalization (Figure S1G) was performed as follows: (1) For each blot, UCP2 and SDHA intensity values for each macrophage subset (UCP2_(MΦ)_, SDHA_(MΦ)_) were divided by the corresponding values from spleen tissue (UCP2_(Sp)_, SDHA_(Sp)_), resulting in UCP2_(MΦ)_/UCP2_(Sp)_ and SDHA_(MΦ)_/SDHA_(Sp)_. (2) The relative protein amount (*I*_rel_) was calculated as *I*_rel_ = ITP/IHK, where ITP is the intensity of the target protein, and IHK is the intensity of the normalization control (SDHA, β‐actin, or α‐tubulin). Therefore, UCP2_(MΦ)_/UCP2_(Sp)_ values were divided by SDHA_(MΦ)_/SDHA_(Sp)_ values to correct for mitochondrial loading. (3) UCP2/SDHA ratios for LPS‐MΦ and IL4‐MΦ samples were normalized to the ratio of resting MΦ samples.

### 2.5 RNA Isolation and Quantitative PCR Analysis

Gene expression analysis was performed as previously described [31]. In brief, total RNA was isolated from BMDMΦs using TRI‐Reagent (RT111, Molecular Research Center, Ohio, USA) following the manufacturer's instructions. cDNA synthesis was performed using the high‐capacity cDNA reverse transcription kit (AM1722, Applied Biosystems, California, USA), according to the manufacturer's instructions. For gene expression analysis with qRT‐PCR, primers were designed to span exon–exon junctions using NCBI Primer‐BLAST. PCR product sizes were kept in the range of 80–150 bp to ensure high primer efficiency. Only primers with amplification efficiencies between 90% and 105%, verified through serial dilution of cDNA, were used. Amplicon sizes were validated on polyacrylamide gels.

Quantitative reverse transcription PCR (qRT‐PCR) was performed on a qTower384 real‐time PCR system (Analytik Jena GmbH+Co.) using Luna master mix (M3003, New England Biolab Company). Plates were loaded in triplicate using 1:1 diluted cDNA, and amplification was performed at 62°C annealing temperature. Murine ribosomal protein L4 (RPL4) was used as a housekeeping gene. CT values for *Ucp2*, hypoxia‐inducible factor 1α gene (HIF1A), Egl Nine homolog 1 gene (*Egln1*), and *Rpl24* were determined for each sample. Primer sequences are listed in Table S2. UCP2 mRNA expression was normalized to mRPL4 levels.

### 2.6 Determination of Oxygen Consumption Rate (OCR) and Extracellular Acidification Rate (ECAR)

BMDMΦs were seeded in Seahorse 96XFe plates at a density of 80,000 cells per well and incubated overnight in RPMI medium supplemented with 2 mM glutamine, 10 mM glucose, 1 mM pyruvate, 10% FBS, and 1% P/S. For nutrient starvation experiments, the entire plate was washed with PBS and refilled with fresh media (control or nutrient‐starved). Polarization was performed for four and 18 h as previously described.

One hour before the experiment, the media were replaced with Seahorse XF RPMI assay medium (103681, Agilent), supplemented with the specified concentrations of glucose (103577, Agilent), glutamine (103279, Agilent), and pyruvate (103578, Agilent). OCR and ECAR were measured in parallel using an XFe96 extracellular flux analyzer (Agilent). The compounds for the Seahorse XF Cell Mito Stress Test ‐ 4.5 µM oligomycin (75351, Sigma Aldrich), 4.5 µM carbonyl cyanide4‐(trifluoromethoxy)phenylhydrazone (FCCP, C2920, Sigma Aldrich), 2.5 µM rotenone (557368, Sigma Aldrich), and 1.25 µM antimycin A (A8674, Sigma Aldrich)—were added according to the manufacturer's instructions. Each independent experiment was performed on a new plate. The minimal number of wells per condition for each experiment was five for basal measurements and three for the Mito Stress Test and mitochondrial nutrient usage inhibition analysis. OCR and ECAR values for each well were normalized to the total protein concentration of the cells seeded in that well.

### 2.7 Proliferation Assay

BMDMΦs or RAW 264.7 cells were seeded in 6‐well plates at a density of 25 × 10^5^ cells per well and incubated overnight in control media. For nutrient shortage experiments, the entire plate was washed with PBS and refilled with fresh media (control or nutrient‐deprived), followed by polarization as described previously. Cell proliferation was monitored using the Incucyte S3 Live‐Cell Analysis System (Sartorius Lab Instruments GmbH & Co., Göttingen, Germany). This real‐time live‐cell analysis assay allows continuous monitoring and quantification of cell growth over time. The software was programmed to take 16 pictures every 2 h over a period of 1 to 2 days.

### 2.8 RNA Sequencing Data Processing

Raw reads were analyzed with a Nextflow v23.10.1 workflow, which included the following steps: analysis of read quality, alignment of the reads, and transcript quantification [32]. The read quality was verified by FastQC v0.12.1 with default parameters, followed by index building and read alignment against the GRCm39 reference genome with STAR v2.7.11, and using the Gencode release M34 GTF file as the annotation parameter [33] (https://www.bioinformatics.babraham.ac.uk/projects/fastqc). The following parameters were specified for STAR alignment: –outSAMtype BAM SortedByCoordinate –outSAMunmapped Within. Next, an indexed transcriptome was built with Gencode Mouse Release M34, followed by quantification of transcripts. Indexing and quantification were performed using Salmon v1.10.3 0.3 [34] with default parameters for index building. For quantification, the following parameters were specified: –gcBias –validateMappings. All reference datasets were obtained from Gencode (https://www.gencodegenes.org/mouse). MultiQC was used to aggregate quality reports [35]

### 2.9 Unsupervised Clustering of the TRM Gene Count Matrices

K‐Means clustering was performed with the R stats package v4.3.1 [36]. The evaluation of clustering quality was assessed using the fviz_nbclust function from the R factoextra package v1.0.7 [37], with the following methods: elbow method, silhouette method, and gap statistic. To examine gene expression differences between samples for Ucp2, a CPM gene activity matrix was used. Gene‐specific changes based on Z‐score values (calculated as the difference between a specific sample's gene expression level and the mean divided by the standard deviation) were visualized with the pheatmap package (Pheatmap: pretty heatmaps version 1.0.12 from CRAN) [38].

### 2.10 Statistics

Gene and protein expression data were analyzed using GraphPad Prism software 7 (GraphPad Software, San Diego, CA, USA). Relative mRNA expression was calculated using the 2^−ΔΔCt^ method. Statistical analysis of the data was performed using one‐way analysis of variance (ANOVA), with the Kruskal–Wallis test for nonparametric data and Dunn's multiple corrections. Protein expression was analyzed with one‐way ANOVA using Dunnett's multiple comparisons test. A significance level was set to 0.05. Significant differences are indicated as follows: *p < 0.05, **p < 0.01, ***p < 0.001.

For quantification of OCR and ECAR analysis, group means and variances were pooled using a weighted fixed‐effects approach based on one‐way analysis of variance (ANOVA), where each group's contribution was weighted by its sample size to compute the overall mean and variance according to standard ANOVA formulas for combined samples. Calculations were performed using an online Java‐based tool (University of Baltimore Statistics Applets: https://home.ubalt.edu/ntsbarsh/business‐stat/otherapplets/Pooled.htm).

## 3 Results

### 3.1 UCP2 Expression in BMDMФs Differentiated From Bone Marrow‐Derived Monocytes

**FIGURE 1:**
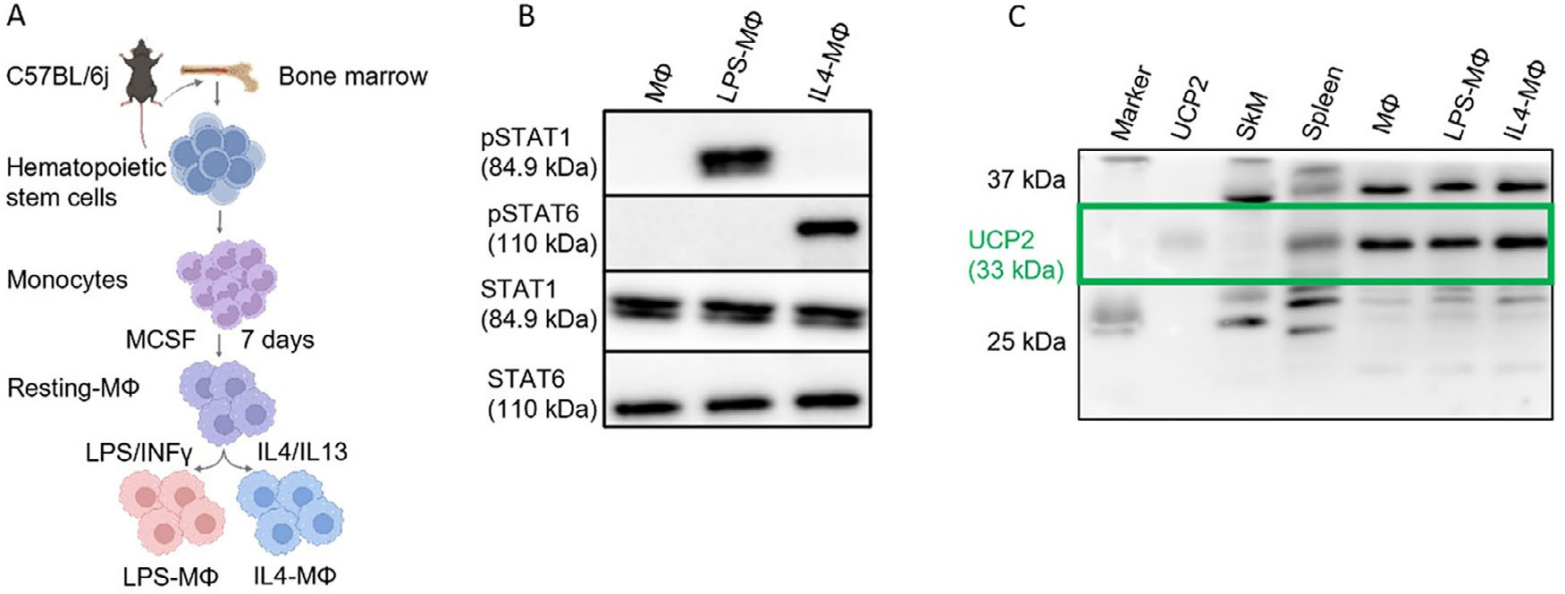
UCP2 abundance in polarized bone marrow‐derived macrophages. (A) Schematic representation of the differentiation and polarization of bone marrow‐derived macrophages into pro‐ and anti‐inflammatory phenotypes. (B) A representative Western blot showing STAT1 and STAT6, as well as their phosphorylated forms (pSTAT1 and pSTAT6), in LPS‐ and IL4‐stimulated MΦ, respectively. (C) A representative Western blot confirming UCP2 expression in pro‐ and anti‐inflammatory macrophages polarized under physiological nutrient levels for 18 h. Recombinant mouse UCP2 (1 ng) and spleen tissue were used as positive controls for UCP2 expression, and skeletal muscle (SkM) tissue was used as a negative control. 20 µg of total cell or tissue protein was loaded per lane.

Bone marrow‐derived monocytes were differentiated into macrophages using macrophage colony‐stimulating factor (MCSF) and then polarized into pro‐inflammatory (LPS‐MΦ) or anti‐inflammatory (IL4‐MΦ) macrophages using LPS/INFγ and IL4/IL13, respectively (Figure 1A). The success of the differentiation was verified by the presence of phosphorylated STAT1 in LPS‐MΦ and phosphorylated STAT6 in IL4‐MΦ after 18 h [39] (Figure 1B).

Immunoblot analysis confirmed the presence of UCP2 protein in all BMDMΦ subsets, similar to bone marrow‐derived monocytes (Figure 1C). To avoid the challenges associated with nonspecific commercial antibodies in UCP2 research, which have led to controversial results in various studies, we used a custom‐designed polyclonal anti‐UCP2 antibody [17] that has been previously validated and applied in independent studies [12, 16, 28, 40]. The specificity of the detected bands was confirmed by positive signals in the applied positive controls (recombinant mouse UCP2, mouse spleen) and their absence in the negative control (skeletal muscle, SkM) (Figure 1C).

### 3.2 Evaluation of UCP2 Protein Levels in Differently Polarized Macrophages

**FIGURE 2:**
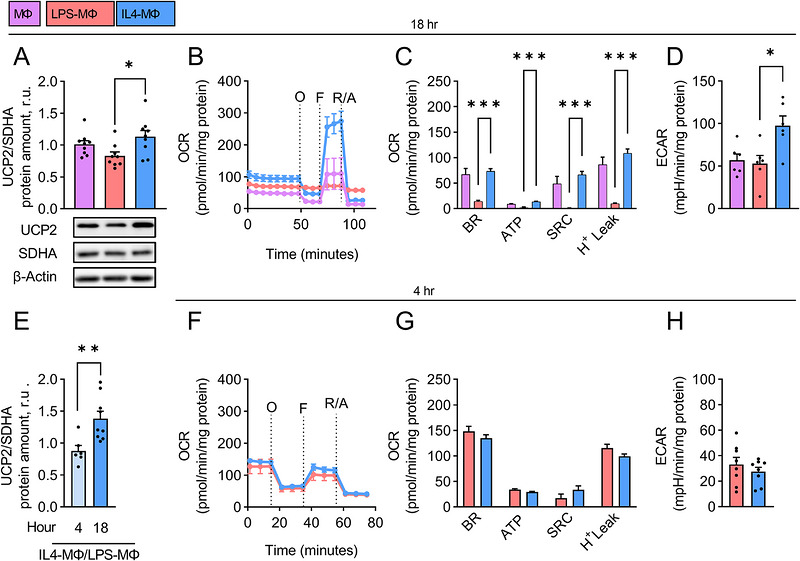
Correlation of polarization state and metabolism of BMDMΦs with UCP2 protein levels under physiological nutritional conditions. (A) Representative WB of UCP2, SDHA, and β‐actin with quantification analysis of UCP2/SDHA (*N* = 9). (B) Representative oxygen consumption rate (OCR) analysis, (C) Quantification of OCR‐derived parameters, and (D) extracellular acidification rate (ECAR) (*N* = 5) in MΦs (left untreated), LPS‐MΦs, and IL4‐MΦs. Polarization was performed for 18 h under physiological macronutrient levels. (E) The ratio of UCP2/SDHA quantification analysis in IL4‐MΦs to the LPS‐MΦs following 4 and 18 h of polarization under physiological nutrient conditions. (F) Representative OCR, (G) quantification of OCR‐derived parameters, and (H) ECAR (*N* = 4) in MΦs, LPS‐MΦs, and IL4‐MΦs. Polarization was performed for four hours at physiological nutrient levels. 20 µg of isolated total protein from each group was loaded per lane. Data are presented as mean ± SEM. *p < 0.05, **p < 0.01, ***p < 0.001. O, oligomycin; F, FCCP; R/A, rotenone/antimycin. BR, basal respiration; SRC, spare respiratory capacity.

To evaluate whether UCP2 protein levels vary upon macrophage polarization under physiological nutrient conditions (5.5 mM glucose, 2 mM glutamine, and 1 mM pyruvate), we performed immunoblot analysis of BMDMΦs at different time points of polarization. The results showed a decrease in UCP2 levels in LPS‐MΦ compared with IL4‐MΦ (Figure 2A). Notably, UCP2 protein levels in IL4‐MΦ and LPS‐MΦ did not differ significantly during the first 4 h of polarization (Figure 2E). The ratio of succinate dehydrogenase (SDHA) to actin, used as a control for mitochondria amount, remained unchanged (Figure S1A).

OCR analysis showed a flat curve for LPS‐MΦ (Figure 2B), consistent with the decreased basal respiration of LPS‐MΦ compared with IL4‐MΦ (Figure 2C). Glycolytic lactate production, as reflected by the ECAR, was higher in IL‐4‐MΦs than in LPS‐MΦs (Figure 2D). In general, IL4‐MΦs showed increased mitochondrial activity, as spare respiratory capacity, mitochondria‐related ATP production, and proton leak were much higher than in LPS‐MΦs (Figure 2C).

Both LPS‐MΦ and IL4‐MΦ responded similarly to respiratory chain (RC) inhibitors, showing identically respiring mitochondria 4 h postpolarization (Figure 2F). Basal respiration, ATP production, and proton leak were comparable between the two phenotypes (Figure 2G). The ECAR was also similar between the two subsets at the time of macrophage polarization initiation (Figure 2H).

In line with literature [23], UCP2 mRNA and protein levels were differently altered upon 18 h of polarization, as analysis of nonpolarized and polarized BMDMΦs under physiological nutrient conditions showed no regulation of UCP2 mRNA levels relative to *mRpl4* (Figure S2A).

The proliferation rate of nonpolarized and polarized BMDMs was assessed over 48 h using an Incucyte scanning device. The results showed no increase in proliferation rates in any phenotype of BMDMΦs (Figure S2D), suggesting that macrophages do not proliferate once they are polarized into the inflammatory and anti‐inflammatory subsets. However, this result is only partially supported by other studies, as some studies on anti‐inflammatory macrophages have shown that their proliferation is regulated by protein 53 (p53) [41, 42].

### 3.3 Comparison of UCP2 Levels and Respiratory Parameters in Differently Polarized Macrophages in the Absence of Glucose

To investigate the impact of glucose deprivation on UCP2 expression in macrophages, we performed immunoblot analysis on polarized macrophages under glucose‐free conditions. The validation of macrophage polarization under different nutritional conditions is presented in Figure S1H.

**FIGURE 3:**
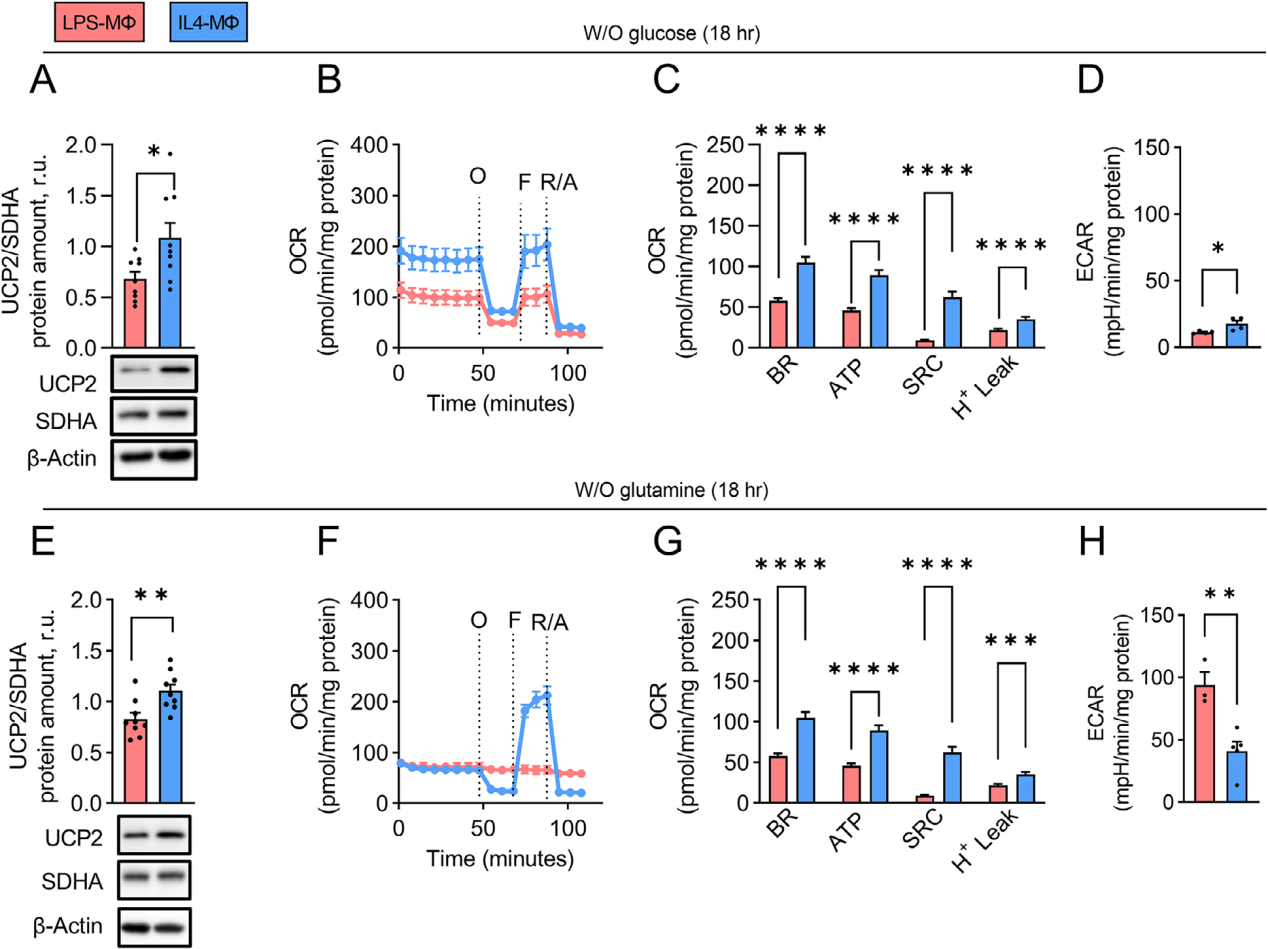
Correlation of polarization state and metabolism of BMDMΦs with UCP2 protein levels in the absence of glucose (A–D) or glutamine (E–H). (A) Representative WB of UCP2, SDHA, and β‐actin with quantification analysis of UCP2/SDHA (*N* = 9), (B) representative OCR, (C) Quantification of OCR‐derived parameters, and (D) ECAR (*N* = 5) in LPS‐ and IL4‐stimulated MΦs. Polarization was performed for 18 h in a medium without glucose. (E) Representative WB of UCP2, SDHA, and actin with quantification analysis of UCP2/SDHA (*N* = 9), (F) representative OCR, (G) quantification of OCR‐derived parameters, and (H) ECAR (*N* = 5) in LPS‐ and IL4‐stimulated MΦs. Polarization was performed for 18 h in a medium without glutamine. Glucose concentration was 5.5 mM. 20 µg of isolated total protein from each group were loaded per lane. Data are presented as mean ± SEM, *p < 0.05, **p < 0.01, ****p < 0.0001. O, oligomycin; F, FCCP; R/A, rotenone/antimycin. BR, basal respiration; SRC, spare respiratory capacity.

The results showed a decrease in UCP2 levels in LPS‐MΦ compared with IL4‐MΦ after 18 h of polarization in the absence of glucose (Figure 3A), although no significant difference was observed after 4 h (Figure S3A).

In the absence of glucose, mitochondria from LPS‐MΦ remained respiring and responded to inhibitors of respiratory complexes (Figure 3B). However, these respirating mitochondria exhibited a lower level of all OCR parameters in comparison to IL4‐MΦs (Figure 3C). Likewise, ECAR was lower in LPS‐MΦ than in IL4‐MΦ (Figure 3D). The absence of glucose led to reduced lactate production, which in turn caused lower ECAR in MΦs polarized for 18 h without glucose. After 4 h polarization, both basal respiration and other respiratory parameters, including ATP production, respiratory capacity, and proton leak, were similar between both phenotypes (Figure S3B). LPS‐MΦs also exhibited ECAR levels similar to IL‐4‐MΦs after 4 h (Figure S3C), consistent with the results from the 18 h incubation.

### 3.4 Comparison of UCP2 Levels and Respiratory Parameters in Differently Polarized Macrophages in the Absence of Glutamine

**FIGURE 4:**
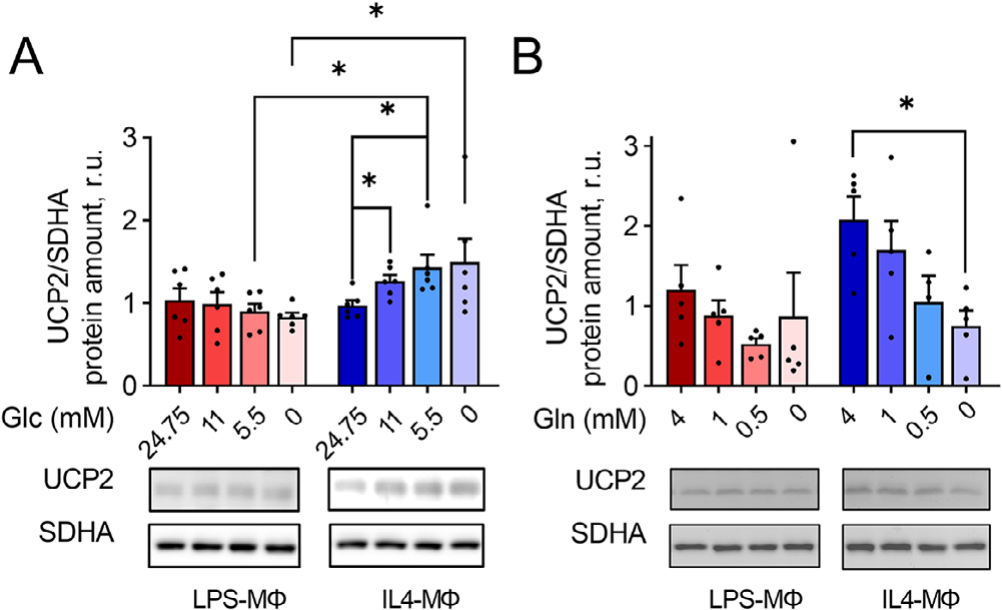
UCP2 protein levels under different glucose and glutamine concentrations. Representative Western blot (WB) and quantification analysis of UCP2/SDHA in LPS‐MΦs and IL4‐MΦs, which were polarized for 18 h under the following conditions: (A) 24.75, 11.1, 5.5, and 0 mM glucose in the presence of 2 mM glutamine, or (B) 4, 1, 0.5, and 0 mM glutamine in the absence of glucose (*N* = 6). Twenty micrograms of total protein isolated from each group were loaded per lane. Data are presented as mean ± SEM; *p < 0.05.

Immunoblot analysis of macrophages polarized in the absence of glutamine revealed lower levels of UCP2 in LPS‐MΦs than in IL4‐MΦ after 18 h incubation (Figure 3E) and 4 h incubation (Figure S3E). OCR analysis of LPS‐MΦs cultured in glutamine‐free conditions revealed a flat curve (Figure 3F), similar to those observed under physiological nutrient conditions. However, the OCR profile of LPS‐MΦs did not display a flat curve in the absence of glucose (Figure 3B). Therefore, we questioned whether the glycolytic product lactate might account for the lack of RC activity in LPS‐MΦs [43, 44]. To investigate this possibility, we again performed OCR analysis on LPS‐MΦs polarized for 18 h in the absence of glucose. Injecting 25 mM lactate, followed by RC inhibitors, showed that the mitochondria in the LPS‐MΦs remained respiring, similar to the control macrophages (Figure S3D). This result excludes lactate as an explanation for the observed effects.

Basal respiration and all other respiratory parameters, including ATP production, respiratory capacity, and proton leak, were significantly lower in LPS‐MΦ compared with IL4‐MΦ after 18 h (Figure 3G) in the absence of glutamine. Only basal respiration and proton leak reached higher levels in IL4‐MΦ than in LPS‐MΦ after 4 h (Figure S3F). ECAR was higher in LPS‐MΦ than in IL4‐MΦ after both 18 h (Figure 3H) and 4 h (Figure S3G).

### 3.5 Comparison of UCP2 Gene Levels in Differently Polarized Macrophages

UCP2 mRNA levels did not differ among different subsets of macrophages under glutamine or glucose deprivation (Suppl. Figure S2B,C). Neither IL4‐MΦ nor LPS‐MΦ exhibited proliferative activity under these conditions (Figure S2E,F). Gene expression analysis of *Ucp2* in BMDMs did not correlate with UCP2 protein levels under different nutritional conditions or polarization states. This discrepancy aligns with previous studies on UCP2 under glucose deprivation [28], rotenone‐induced oxidative stress [45], and LPS injection [23] in pulmonary tissues. This phenomenon is often attributed to translational and posttranslational regulation of UCP2 [23, 46, 47]. Additionally, UCP2 has a half‐life of less than 30 min [48] and is not degraded by the cytosolic proteasome [49]. However, LPS has been shown to induce proteasomal degradation of UCP2 [50]. To address these discrepancies and test whether MG132, an inhibitor of proteasome degradation, could prevent the reduction in UCP2 protein levels in LPS‐MФs, we exposed the cells to MG132 for 2 h  before 18 h polarization. Reduced degradation of the inhibitor of nuclear factor kappa B alpha (IκBα) in the presence of MG132 indicates that proteasomal activity is partially inhibited, though not completely (Figure S2G). Despite this partial inhibition, we observed no increase in *Ucp2* levels in the MG132‐treated group compared with the untreated control group (Figure S2G). However, since the inhibition was incomplete, we cannot rule out the possibility that proteasomal degradation is responsible for the reduced *Ucp2* levels observed in LPS‐MΦs.

### 3.6 Comparison of UCP2 Expression and Respiratory Parameters in RAW264.7 Cells Under Different Nutrient Conditions

As additional control for our experiments, we polarized the immortalized macrophage‐like cell line RAW264.7 into inflammatory and anti‐inflammatory phenotypes under different concentrations of glucose, glutamine, and pyruvate. In contrast to primary BMDMΦs, UCP2 levels in RAW264.7 cells remained unchanged across all phenotypes. However, RAW264.7 cells were sensitive to glutamine deprivation regardless of polarization state and expressed significantly lower levels of UCP2 in the absence of glutamine, though SDHA levels remained unchanged (Figure S4A–G). LPS‐RAW264.7 cells exhibited a reduced proliferation rate compared with IL4‐RAW264.7 cells under physiological nutrient conditions and after 12 h of glucose deprivation (Figure S4H–J). This difference can be attributed to the proliferative, immortalized nature of RAW264.7 cells, in contrast to the nonproliferative behavior of BMDMΦs (Figure S2D–F).

Overall, these findings suggest UCP2 levels are lower in LPS‐MΦs than in IL4‐MΦs, regardless of glucose or glutamine availability. However, the mitochondrial respiratory profile of LPS‐MΦs is strongly influenced by the presence of glucose. The reduction in UCP2 levels in LPS‐activated macrophages cannot be fully attributed to proteasomal degradation, suggesting the involvement of additional regulatory mechanisms, likely at the translational level. Furthermore, a comparison of primary and immortalized macrophages reveals that UCP2 levels in BMDMΦs correlate more closely with mitochondrial respiration. In contrast, UCP2 expression in RAW264.7 cells largely depends on glutamine availability [12, 28].

### 3.7 Investigation of UCP2 Protein Levels in BMDMΦs at Nonphysiological Levels of Glucose and Glutamine

To investigate whether pathologically high concentrations of glucose (as seen in patients with metabolic syndrome [51])  or glutamine modulate UCP2 levels in BMDMs, polarized and nonpolarized BMDMΦs were incubated with various concentrations of (i) glucose (0–25 mM) at a constant concentration of glutamine (2 mM) (Figure 4A) or (ii) glutamine (0–4 mM) without glucose for 18 h (Figure 4B) or 2 or 4 h (Figure S5).

Immunoblot analysis of BMDMΦs revealed a significant increase in UCP2 levels in IL4‐MΦ, but not in LPS‐MΦ, when incubated with 5.5 and 11 mM glucose compared with 24.75 mM glucose after 18 h of incubation (Figure 4A). Decreasing the glutamine concentration reduced UCP2 expression in IL4‐MΦ after 18 h of incubation (Figure 4B). UCP2 protein levels remained unchanged under different doses of glucose and glutamine after 2 and 4 h (Figure S5C,D,G,H).

Together, these results demonstrate that anti‐inflammatory macrophages adjust their UCP2 levels based on glucose availability. This suggests that these cells have a highly flexible metabolism that depends on nutrient availability and that adjusting UCP2 protein levels is part of their metabolic decision‐making process.

### 3.8 Investigation of UCP2 Levels in the Absence of Pyruvate

It has been proposed that in neuroblastoma cells, UCP2 transports C4 metabolites from the mitochondrial matrix to the cytosol during glucose shortage, providing substrates for conversion into pyruvate, which in turn fuels the TCA cycle [28]. Consistent with this, previous RNA sequencing data from K562 UCP2 knockout cells identified pyruvate kinase R/L (PKRL) as the gene with the highest fold change compared with control K562 cells [12].

**FIGURE 5:**
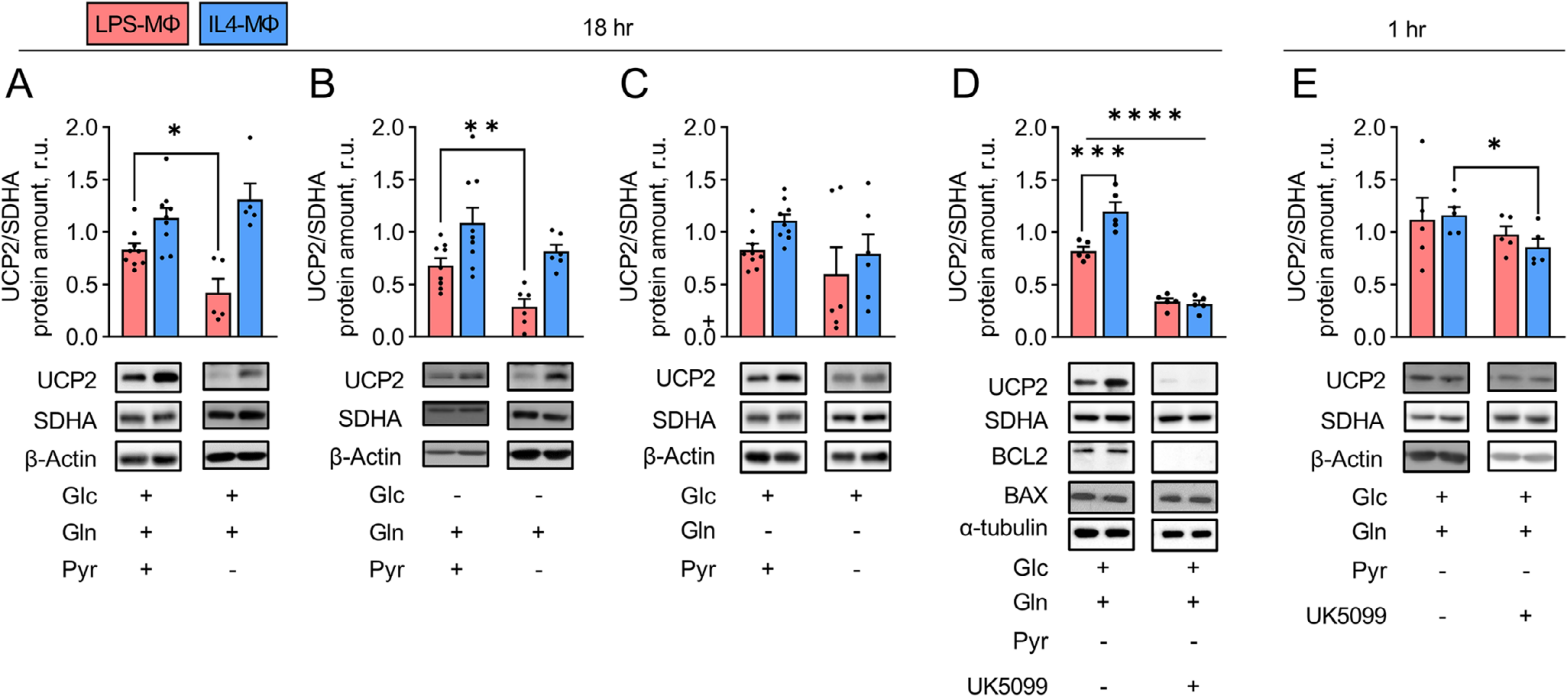
UCP2 levels in the absence of pyruvate or after blocking its uptake into the mitochondria. Representative WBs of UCP2, SDHA, β‐actin, Bcl2, BAX, and α‐tubulin and quantification analysis of UCP2/SDHA in LPS‐MΦs and IL4‐MΦs. Polarization was performed for 18 h under physiological nutrient conditions vs. absence of pyruvate (A), absence of glucose vs absence of glucose and pyruvate (B), absence of glutamine vs. absence of glutamine and pyruvate (*N* = 6–9) (C), and blocking of the mitochondrial pyruvate carrier with UK5099 for overnight (D) and 1 h (E) (*N* = 5). 20 µg of isolated total protein from each group was loaded per lane. Data are presented as mean ± SEM, *p < 0.05, **p < 0.01, ***p < 0.001, ****p < 0.0001. Glc, glucose; Gln, glutamine; Pyr, pyruvate.

Building on this, we examined the impact of pyruvate levels on UCP2 expression in different macrophage subsets. Pyruvate was excluded from the media to create three distinct nutritional conditions: no pyruvate, no glucose/no pyruvate, and no glutamine/no pyruvate. Immunoblot analysis of LPS‐MΦ showed a significant reduction in UCP2 levels in the absence of pyruvate (Figure 5A) and when both pyruvate and glucose were excluded (Figure 5B). This effect depended on glutamine availability, as UCP2 levels remained unchanged when pyruvate was removed in the absence of glutamine (Figure 5C). The ratio of SDHA to ß‐actin remained roughly the same under all experimental conditions (Figure S6A–C).

We also investigated whether blocking pyruvate entry into mitochondria with UK5099, a selective inhibitor of the mitochondrial pyruvate carrier (MPC), affects UCP2 levels. As shown in Figure 5D, BMDMΦs exhibited downregulation of UCP2 while mitochondrial SDHA, OGDH, and VDAC remained unchanged (Figure S6D). α‐Tubulin levels decreased due to the lack of pyruvate metabolism in the mitochondria. No Bcl2 was detected in macrophages, suggesting apoptosis occurred after 18 h of incubation with 10 µM UK5099. We hypothesized that prolonged treatment may induce apoptosis, so we reduced the incubation time to one hour using 50 µM UK5099. This shorter treatment resulted in decreased UCP2 levels in IL4‐MΦ compared with the control group (Figure 5E). Taken together, these experiments highlight pyruvate as a critical nutrient for mitochondrial respiration and macrophage survival. They also confirm that reduced UCP2 levels in LPS‐MΦ are primarily due to pyruvate being converted to lactate rather than being transported into the mitochondria, fueling the TCA cycle.

### 3.9 Levels of the UCP2 Protein in Anti‐Inflammatory Macrophages Under CoCl_2_‐Induced Hypoxia Conditions

**FIGURE 6:**
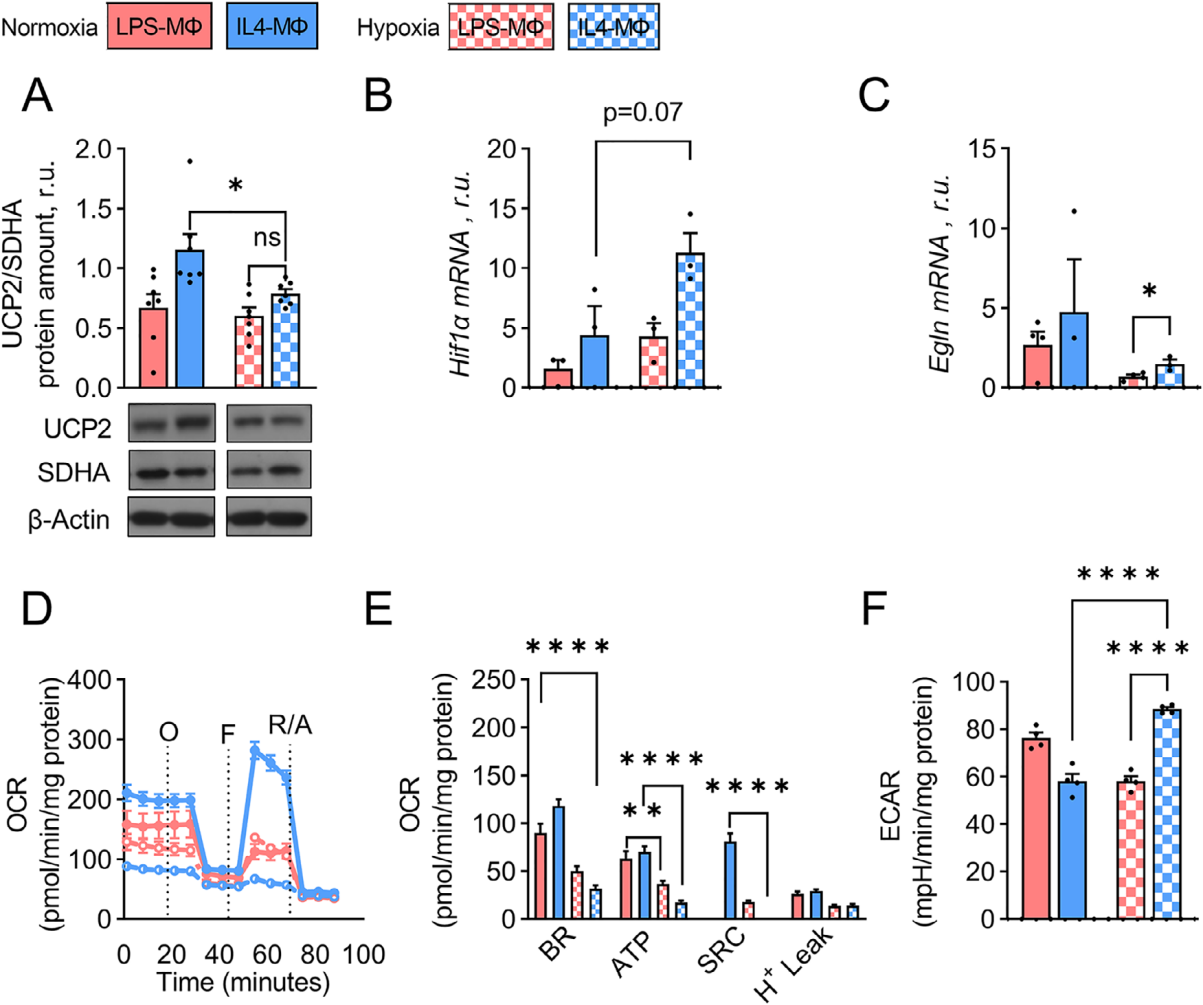
UCP2 levels and metabolism of BMDMΦs under hypoxic‐like conditions. (A) Representative WB and quantification analysis of UCP2/SDHA in LPS‐ and IL4‐stimulated MΦs after overnight polarization and incubation under physiological nutrient conditions in the absence (normoxia) or presence of CoCl_2_ (hypoxia‐like conditions). (*N* = 7). (B) QRT‐PCR analysis of Hif1α (B) and Egln1 (C) genes with mitochondrial ribosomal protein L4 (RPL4) as mitochondrial reference gene in LPS‐MΦs and IL4‐MΦ (*N* = 4). (D) Representative OCR, (E) Quantification of OCR‐derived parameters, and (F) ECAR (*N* = 4) in LPS‐MΦs and IL4‐MΦs. Polarization was performed for 18 h under normoxia or hypoxia‐like conditions at physiological nutrient levels. 20 µg of isolated total protein from each group were loaded per lane. Data are presented as mean ± SEM, *p < 0.05, **p < 0.01, ****p < 0.0001. O, oligomycin; F, FCCP; R/A, rotenone/antimycin. BR, basal respiration; SRC, spare respiratory capacity. The amount of mRNA is presented in relative units to RPL4, which was used as a housekeeping gene and calculated using the 2^−ΔΔCt^ method.

Since lactate production is a hallmark of oxygen‐deprived conditions, which can also be triggered by LPS [52], we investigated whether we could mimic the observed effects on UCP2 levels in LPS‐MΦ in another model: BMDMΦs exposed to hypoxic conditions. Therefore, macrophages were cultured and polarized in a CoCl_2_‐induced, hypoxia‐mimicking environment for 18 h. Immunoblot analysis revealed that the hypoxia‐mimicking environment reduced UCP2 levels in IL4‐MΦ compared with normoxic conditions, resulting in UCP2 expression levels comparable to those observed in LPS‐MΦ (Figure 6A). However, no changes in UCP2 levels were observed in LPS‐MΦ under hypoxic conditions, indicating the maximal downregulation effect of LPS alone.

SDHA protein levels remained unchanged under hypoxia‐mimicking conditions (Figure S7). IL‐4‐treated macrophages exhibited a trend to increase Hif1a mRNA and to decrease Egln1 mRNA under these conditions (Figure 6B,C). Extracellular flux analysis revealed a decrease in basal OCR, ATP production, and spare respiratory capacity in IL4‐MΦs under hypoxia compared with normoxia (Figure 6D,E). Conversely, ECAR of IL4‐MΦs increased upon exposure to the hypoxia‐mimicking environment (Figure 6F). These results strengthen the previously postulated mechanism of UCP2's dependency on pyruvate, lactate, and oxygen. Our findings suggest that hypoxic conditions induced by CoCl_2_ stabilize HIF‐1α while maintaining EGLN1/PHD2 (egl‐9 family hypoxia inducible factor 1/prolyl hydroxylase domain‐containing protein 2) expression. This leads to the suppression of mitochondrial metabolism, including reduced pyruvate oxidation by pyruvate dehydrogenase (PDH) [53]. Since UCP2 mediates the mitochondrial export of TCA cycle intermediates, such as malate, oxaloacetate, and aspartate [27], the reduced mitochondrial flux under these conditions likely decreases the demand for UCP2 activity. Consequently, the lower levels of UCP2 protein observed in IL‐4 macrophages exposed to CoCl_2_ may reflect a coordinated metabolic adaptation to diminished oxidative metabolism and TCA cycle activity during hypoxia.

### 3.10 Variability in UCP2 mRNA Levels Among Tissue‐Resident Macrophages

**FIGURE 7:**
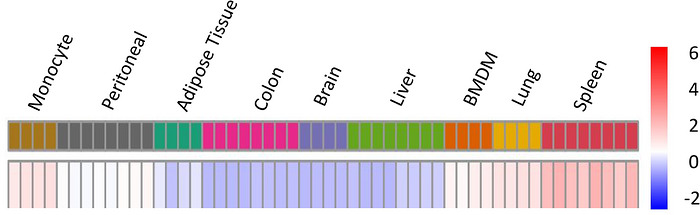
RNA sequencing analysis of tissue‐resident macrophages. Heatmap illustration of UCP2 mRNA Z‐score in different tissue‐resident macrophages (TRMs), resulting from RNA sequencing analysis of TRMs from 4 to 8 different mice. The colors represent the tissue of origin of the macrophages, with each mouse contributing a different column.

To place the data in a broader context and highlight the significance of our findings, we analyzed whether *Ucp2* expression varies across different murine TRMs. RNA sequencing analysis revealed that TRMs from the spleen exhibited the highest Z‐score for UCP2 mRNA (Figure 7), followed by blood monocytes, bone marrow‐derived macrophages (BMDMs), and lung‐associated macrophages.

Peritoneal macrophages displayed a relatively lower Z‐score, indicating a basal level within the grading scale. Interestingly, macrophages derived from adipose tissue, brain, colon, and liver showed negative Z‐scores for UCP2 mRNA (Figure 7). These results demonstrate significant variation in UCP2 mRNA expression across different TRM populations. However, since mRNA levels do not always correlate with protein expression, further studies are needed to assess the distribution of UCP2 protein levels among the TRMs.

## 4 Discussion

**FIGURE 8:**
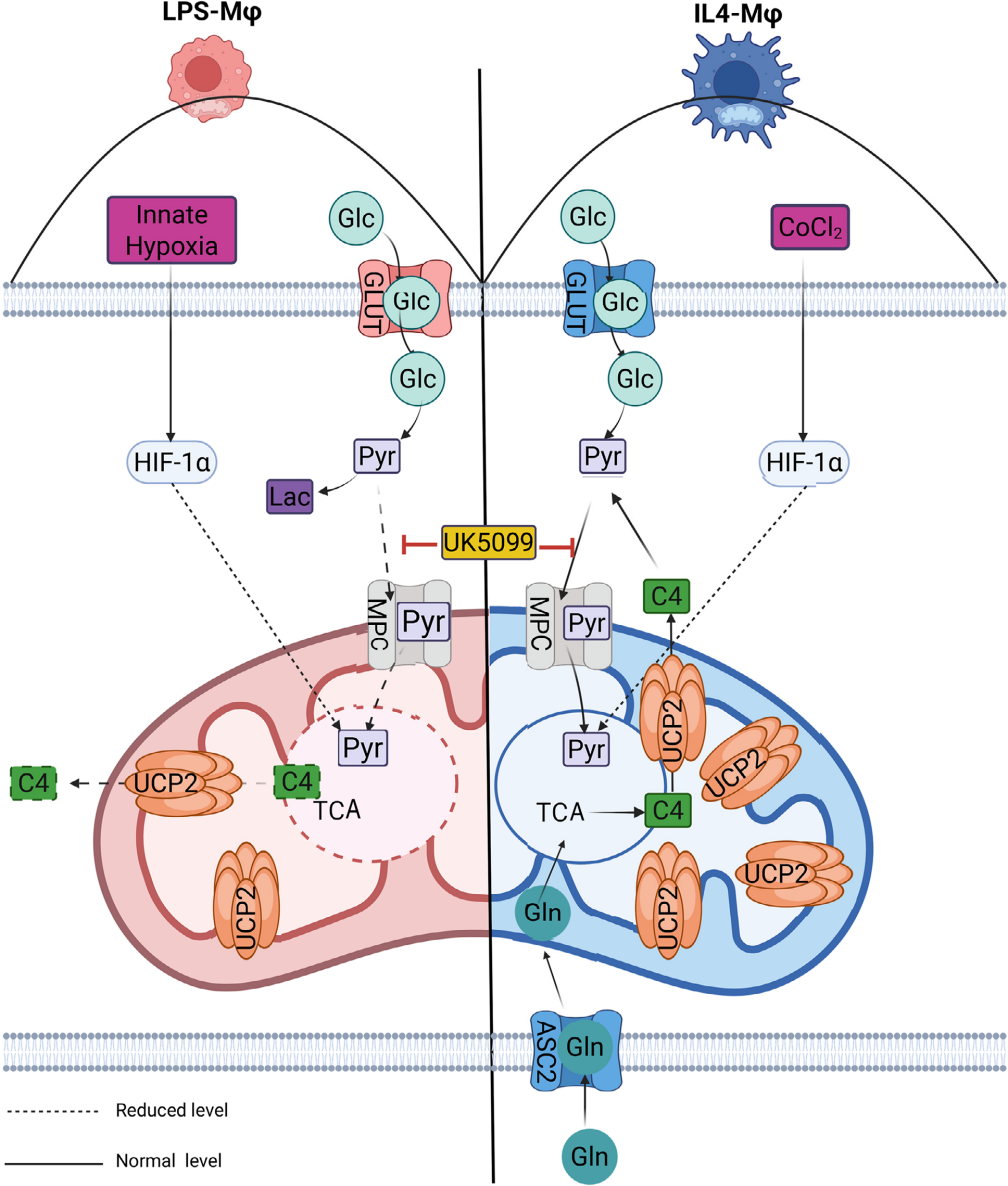
Regulation of UCP2 by available metabolic substrates in macrophage subsets. In LPS‐stimulated macrophages (LPS‐MΦs), glucose uptake leads to increased glycolysis, with pyruvate being preferentially converted into lactate. This results in high lactate production and export, and very little pyruvate enters the mitochondria. Because TCA cycling is limited, fewer C4 metabolites are generated. This leads to a lower need for C4 metabolite transport activity of UCP2, resulting thereby in its lower amounts. In contrast, IL4‐MΦs use more glucose for oxidative metabolism. Here, pyruvate is transported into mitochondria and enters the TCA cycle, resulting in higher C4 metabolite production. UCP2 is proposed to transport these C4 metabolites out of mitochondria. The increased metabolite flux may explain why the higher UCP2 is more expressed in IL4‐MΦs than in LPS‐MΦs. Exported C4 metabolites can be reconverted into pyruvate to sustain TCA cycle activity. In IL4‐MΦs under conditions of low glucose availability, IL4‐MΦs utilize glutamine as an alternative substrate. Glutamine enters the mitochondria and fuels the TCA cycle, thereby increasing C4 metabolite levels. This pathway can contribute to maintaining UCP2‐associated metabolite transport. In both subsets, inhibition of the mitochondrial pyruvate carrier (MPC) with UK5099 blocks mitochondrial pyruvate entry. It results in reduced TCA cycle activity and, consequently, almost no UCP2 expression. Innate hypoxia in LPS‐MΦs, or CoCl_2_‐induced hypoxia‐mimicking conditions, stabilizes HIF‐1α. Elevated HIF‐1α reduces oxygen‐dependent mitochondrial respiration, decreasing the need for UCP2‐mediated metabolite transport and leading to lower UCP2 protein levels in both macrophage types.

The present study demonstrates that, after 18 h of polarization, IL4‐stimulated macrophages exhibit higher UCP2 levels and a higher OCR than LPS‐stimulated macrophages (Figure 8). Furthermore, IL4‐MΦs showed a decrease in UCP2 amount under high (pathological) glucose levels compared with cells cultured under physiological glucose conditions. However, reducing glutamine in the absence of glucose further decreased UCP2 levels. Notably, blocking pyruvate entry into mitochondria for 18 h led to a loss of UCP2 protein in both macrophage phenotypes. Under hypoxia‐mimicking conditions induced by CoCl_2_, IL4‐MΦs showed a significant reduction in UCP2 expression and OCR, which brought UCP2 levels closer to those observed in LPS‐MΦs. These results suggest that the expression of UCP2 is tightly regulated by the metabolic state of the macrophages.

### 4.1 IL4‐MΦs Have Higher UCP2 Levels Than LPS‐MΦ, Which  Correlates With Their OCR

Our study confirmed the presence of UCP2 in macrophages, consistent with previous studies in immune cells [18, 54]. Unlike most in vitro studies that focused only on LPS‐MΦs [19, 21, 55, 56, 57], we directly compared UCP2 levels between LPS‐MΦs and IL4‐MΦ. Our results confirmed that UCP2 levels were higher in mouse IL‐4–activated macrophages than in LPS‐stimulated macrophages, which is in agreement with findings in human primary macrophages [58]. Some inconsistencies reported in the literature, for example, decreased UCP2 levels in peritoneal macrophages after LPS treatment [20, 59, 60] while unchanged UCP2 levels in LPS‐treated microglial cells after 18 h [61], may stem from the use of nonvalidated antibodies or lack of appropriate controls, which can lead to variable UCP2 detection.

The correlation between UCP2 levels and oxygen consumption rate in IL4‐MΦs suggests that UCP2 might support mitochondrial respiration by facilitating the transport of metabolic substrates into the TCA cycle. Further, these findings suggest that the intrinsic metabolism of each macrophage phenotype determines UCP2 levels as a downstream target. While our LPS‐MΦs displayed a flat OCR curve after 24 h of polarization, other studies have reported normal OCR at later time points, which may be due to differences in cell type or experimental timing [58].

The higher ECAR in IL4‐MΦs under physiological conditions suggests active glycolysis, which is linked to their ability to repolarize to a pro‐inflammatory phenotype, as reported by previous studies [6, 62]. Furthermore, IL4‐MΦs exhibited higher ECAR than LPS‐MΦs in the absence of glucose, suggesting a unique metabolic adaptation that merits further investigation. This may reflect IL4‐MΦs’ greater ability to repolarize toward the pro‐inflammatory state when glutamine is available. Conversely, when glutamine was absent but glucose was present, LPS‐MΦs exhibited higher ECAR, consistent with the rapid conversion of pyruvate‐derived glucose to lactate. Thus, the absence of glutamine appears to limit IL4‐MΦ’s ability to repolarize. Overall, these findings highlight the critical role of glucose and glutamine in regulating IL4‐MΦ’s metabolic flexibility and repolarization potential.

### 4.2 UCP2 Levels in IL4‐MΦs are Regulated by the Availability of Metabolic Substrates

In this study, we explored the impact of glucose, glutamine, and pyruvate on UCP2 levels in BMDMΦs for the first time. We found that UCP2 expression was significantly lower in IL4‐MΦs under pathologically high glucose concentrations compared with lower concentrations. This is likely due to elevated glycolysis and reduced mitochondrial respiration. These results suggest that UCP2 is less critical in cells that use less oxidative phosphorylation in their metabolism. At first glance, this appears paradoxical, as UCP2 is predominantly expressed in cells that primarily depend on glycolysis. However, our recent findings indicate that, in these cells, UCP2 plays an important role in regulating mitochondrial oxidative phosphorylation, which provides a logical explanation for its localization to the inner mitochondrial membrane.

Moreover, UCP2 in IL4‐MΦs was sensitive to glutamine availability, supporting previous observations in cell lines such as N18TG2, HT29, and INS‐1, as well as our current results in RAW264.7 cells. Collectively, these results suggest that glutamine plays a key role in regulating UCP2 expression [27, 28, 63]. In contrast, LPS‐MΦs maintained stable UCP2 levels regardless of glucose and glutamine availability, reflecting their dominant glycolytic metabolism [64] and reduced reliance on the TCA cycle [4].

Vozza et al. [27] showed that the oxidation of pyruvate is limited in HepG2 UCP2‐KO cells in the absence of glucose, resulting in reduced accumulation of TCA intermediates in the cytosol. This occurs due to the lack of UCP2 transport activity, which exports TCA intermediates, such as pyruvate, from the mitochondria to the cytosol. Relevant to this, we observed reduced UCP2 levels in the absence of pyruvate in LPS‐MΦs because they predominantly rely on glycolysis, converting a significant portion of pyruvate to lactate rather than transporting it into the mitochondria [3]. Therefore, limited pyruvate availability leads to a reduction in the TCA cycle activity and mitochondrial respiration, which, in turn, lowers the need for UCP2 activity, as reflected by its lower protein level. In contrast, the levels of UCP2 in IL4‐MΦs were found to be independent of exogenous pyruvate, possibly due to alternative sources, such as glutamine or fatty acids, that IL4‐MΦs use to keep the TCA cycle active [65].

The loss of UCP2 after 18 h of blocking pyruvate entry into the mitochondria may result from significantly lower mitochondrial respiration and, consequently, a lower need for the transport function of UCP2. Constant levels of SDHA, VDAC, and OGDH were observed after pyruvate entry into the mitochondria was blocked (Figure S6D). This indicates that key mitochondrial components are unaffected. These proteins remain stable instead, reflecting the maintenance of basic mitochondrial structure, bioenergetic capacity, and number. This stability supports the notion that UCP2 loss primarily reflects metabolic adaptation rather than mitochondrial degradation, which is consistent with the findings of Vozza et al. [27].

### 4.3 Hypoxia‐Mimicking Conditions Suppress Mitochondrial Respiration and Reduce UCP2 Expression

Under hypoxia‐mimicking conditions induced by CoCl_2_, IL4‐MΦs showed reduced UCP2 levels, suggesting that the lower UCP2 levels observed in LPS‐MΦs may stem from LPS‐induced innate hypoxia [52]. In IL4‐MΦs, HIF1‐α stability can inhibit pyruvate dehydrogenase (PDH) activity [66] under hypoxic conditions. Lower PDH activity reduces the pyruvate amount available for conversion to acetyl‐CoA in the TCA cycle and, consequently, mitochondrial respiration. The lower OCR in IL4‐MΦs under hypoxia‐mimicking conditions, as shown in Figure 6D,E, further confirms the reduction in mitochondrial respiration.

Consistent with this, the higher ECAR in IL4‐MΦs compared with LPS‐MΦs under hypoxia‐mimicking conditions (Figure 6F) is due to HIF1‐α stability, which activates the transcription of glycolytic genes [67, 68]. Considering UCP2's proposed role in regulating mitochondrial ROS, its downregulation under hypoxia likely reflects reduced ROS generation and the diminished need for ROS scavenging [69]. This could also explain the lower expression of UCP2 under hypoxia, where less ROS production leads to less ROS scavenging activity by UCP2 and thus less expression of this protein by cells. Thus, lower UCP2 levels observed under hypoxia can be correlated with lower ROS production [69] and diminished mitochondrial respiration (Figure 6D).

Overall, our data showed that pro‐inflammatory macrophages, which exhibit reduced mitochondrial respiration, are less dependent on UCP2. In contrast, anti‐inflammatory macrophages, which rely more on mitochondrial respiration, utilize UCP2 extensively. Pyruvate is a key substrate regulating UCP2 levels, linking glycolysis and mitochondrial respiration. These findings suggest that macrophages dynamically adjust their UCP2 levels to meet their specific metabolic needs.

## Author Contributions

**Jila Nasirzade**: conceptualization, methodology, validation, formal analysis, investigation, writing – original draft, review and editing, visualization. **Felix Sternberg**: methodology, investigation, review and editing, supervision. **Andrea Vogel**: methodology, investigation, review, and editing. **Roko Sango**: methodology, formal analysis, investigation, review, and editing. **Taraneh Beikbaghban**: investigation, review, and editing. **Thomas Kolbe**: methodology, resources, review, and editing. **Thomas Rattei**: resources, review and editing, supervision. **Thomas Weichhart**: resources, review and editing, supervision, and funding acquisition. **Elena E. Pohl**: conceptualization, validation, resources, review and editing, supervision, project administration, funding acquisition. All authors have approved the final version of the manuscript.

## Funding

This study was supported by the Austrian Science Fund (Sonderforschungsbereich F83 10.55776/F8300 to E.E.P. and T.W.).

## Ethics Statement

All animal experiments were approved by the Austrian national authority according to the Animal Experiments Act, Tierversuchsgesetz 2012‐TVG 2012 (ETK‐172/11/2023).

## Conflicts of Interest

The authors declare no conflicts of interest.

## Supporting information




**Supporting File 1**: eji70218‐sup‐0001‐SuppMat.pdf.

**Supporting File 2**: eji70218‐sup‐0002‐SuppMat.pdf.

## Data Availability

All data are available in the main article or the supplementary materials and from the corresponding authors upon reasonable request.
