# Supplementary material for: Pro‐ and Anti‐Inflammatory Macrophages Adjust UCP2 Protein Levels Based on Their Intrinsic Metabolism and Available Metabolites
Source: Eur J Immunol. 2026 Jun 9;56(6):e70218. doi: 10.1002/eji.70218 (PMC13247729; doi:10.1002/eji.70218)
Supplement: Supplementary file 1 — Supporting File 1: eji70218‐sup‐0001‐SuppMat.pdf. [file EJI-56-e70218-s001.pdf]

SUPPLEMENTARY MATERIALS

Pro- and anti-inflammatory macrophages adjust UCP2 protein level based on their intrinsic metabolism and available metabolites

Jila Nasirzade[1], Felix Sternberg[1,2], Andrea Vogel[2], Roko Sango[3,4,5], Taraneh Beikbaghban[1], Thomas Kolbe[6,7], Thomas Rattei[4], Thomas Weichhart[3], Elena E. Pohl[1, *]

[1]Medical Physics and Biophysics, Department of Biological Science and Pathobiology, University of Veterinary Medicine, Vienna, Austria.

[2]Department of Nutritional Sciences, Faculty of Life Sciences, University of Vienna, Vienna, Austria.

[3]Center of Pathobiochemistry and Genetics, Institute of Medical Genetics, Medical University of Vienna, Vienna, Austria.

[4]Centre for Microbiology and Environmental Systems Science, University of Vienna, Vienna, Austria

[5]Doctoral School in Microbiology and Environmental Science, University of Vienna, Vienna, Austria

[6]Laboratory Animal Medicine, Department of Biological Science and Pathobiology, University of Veterinary Medicine, Vienna, Austria

[7]Department of Agricultural Sciences, University of Natural Resources and Life Sciences, Vienna, Austria

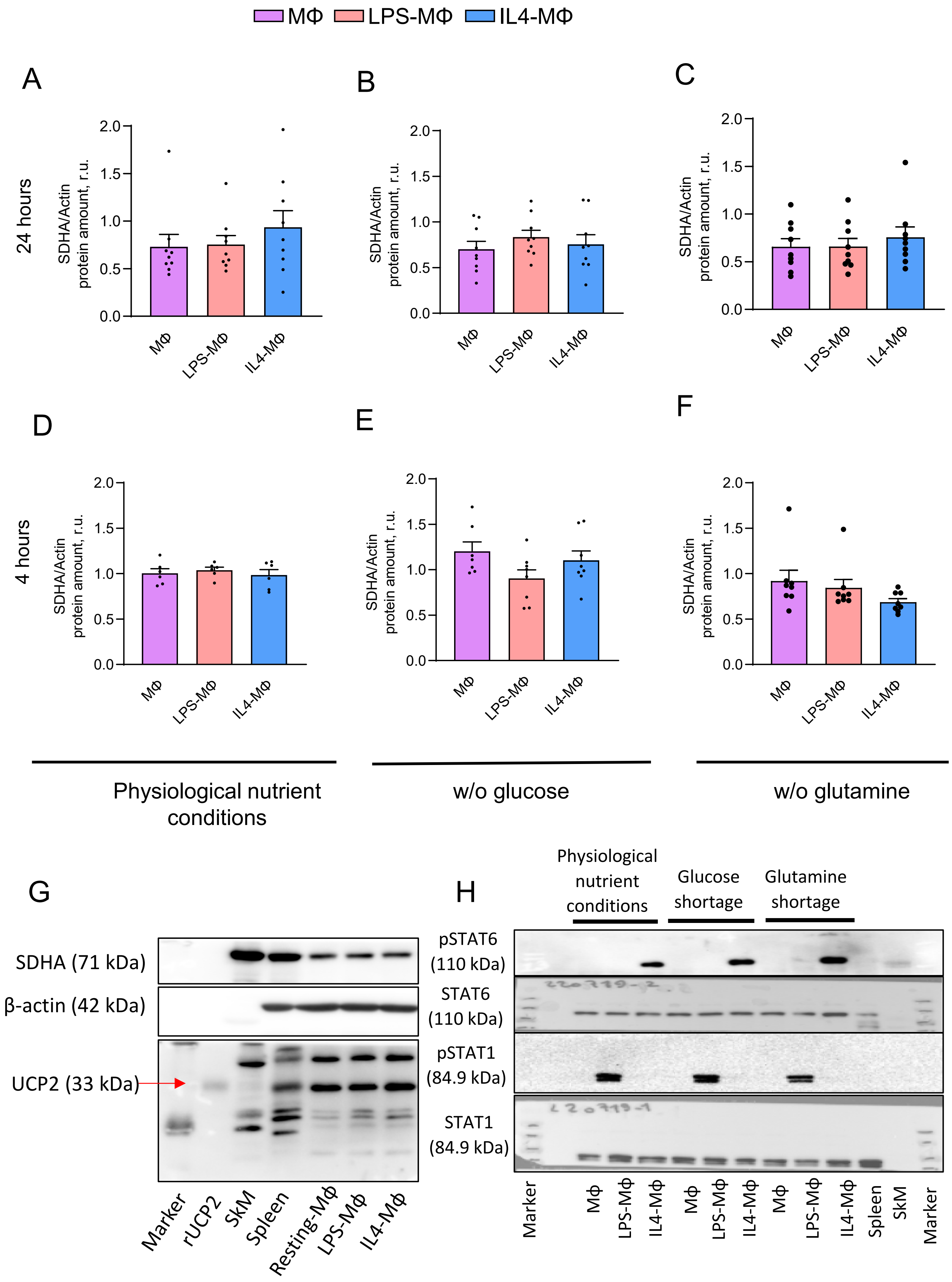

Supplementary Figure 1.

Supplementary Figure 1. Evaluation of SDHA protein levels as a mitochondrial protein control in BMDMΦs under different nutritional challenges.

(A-C) Quantitative analysis of WB of SDHA/actin in MΦs, LPS-MΦs and IL4-MΦs after 24 hours of polarization under conditions mimicking physiological nutrient levels (A), in the absence of glucose (B), and in the absence of glutamine (C).

(D-F) Quantitative analysis of WB of SDHA/actin in MΦs, LPS-MΦs and IL4-MΦs after 4 hours of polarization under conditions mimicking physiological nutrient levels (D), in the absence of glucose (E), and in the absence of glutamine (F). Data are presented as mean values ± SEM, n = 6 - 7.

(G) Band intensity normalization was performed in three steps: First, for each immunoblot, the UCP2 and SDHA intensity values for each macrophage subset (UCP2(MΦ)) were divided by the corresponding UCP2 and SDHA intensity values from the spleen (UCP2(Sp)), which were loaded on the same blot. This resulted in UCP2(MΦ)/UCP2(Sp) values for each macrophage subset on each blot. Second, the UCP2(MΦ)/UCP2(Sp) values were divided by the SDHA(MΦ)/SDHA(Sp) values. Finally, the UCP2/SDHA ratio for the LPS-MΦ and IL4-MΦ samples was normalized against the UCP2/SDHA ratio of the resting MΦ samples. rUCP2, recombinant UCP2.

(H) Validation of macrophage polarization under different nutritional conditions.

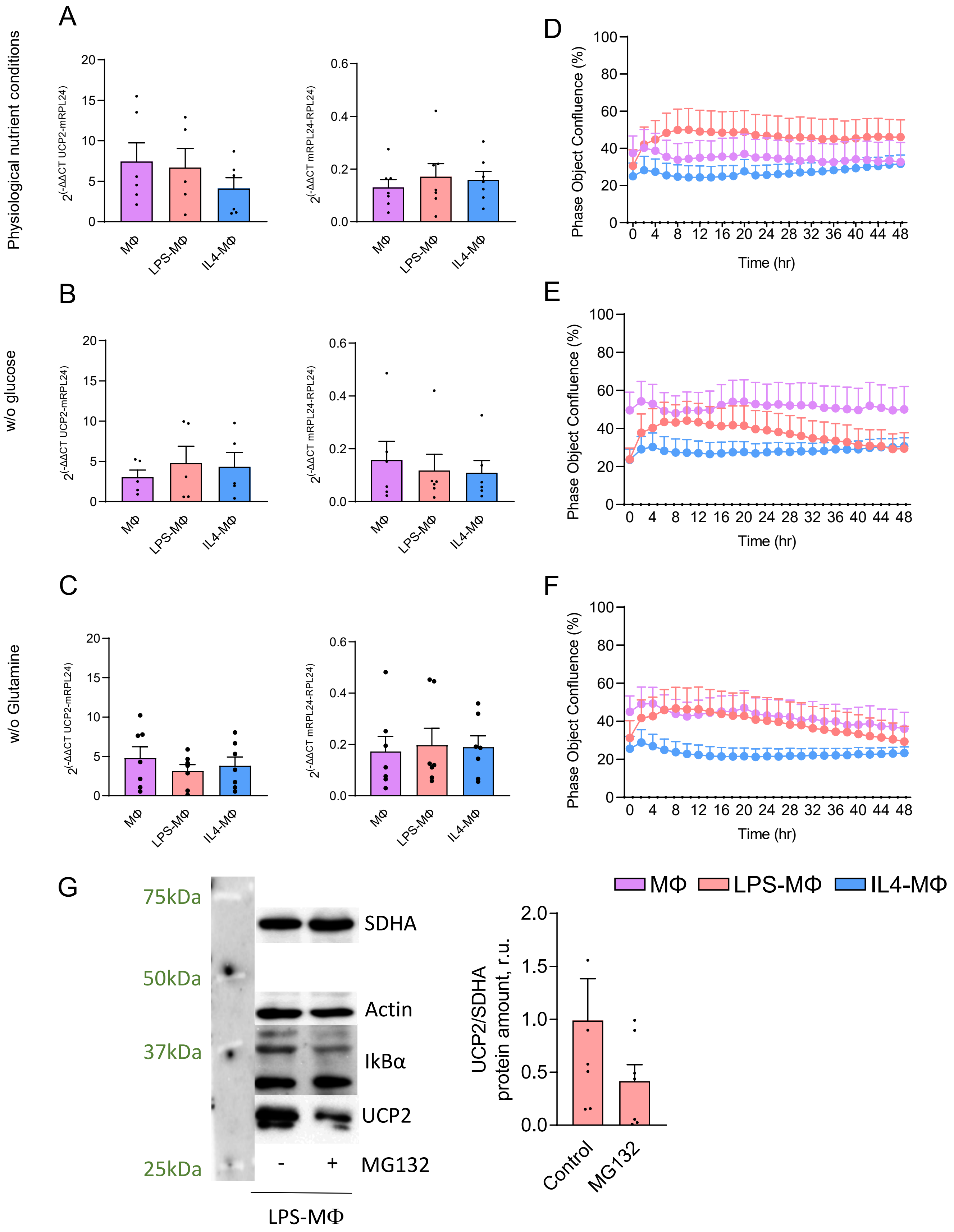

Supplementary Figure 2.

Supplementary Figure 2. Evaluation of UCP2 gene expression and proliferation of BMDMΦs under different nutritional challenges.

(A-C) The BMDMΦs were either unpolarized (MΦ) or polarized using LPS (LPS-MΦ) or IL4 (IL4-MΦ) under the following conditions: (A) under conditions mimicking physiological nutrient levels, (B) in the absence of glucose, and (C) in the absence of glutamine. All conditions were maintained for 18 hours. Then, total RNA was isolated. QRT-PCR analysis of the *Ucp2* gene was performed using the mitochondrial ribosomal protein L24 (m*Rpl24*) as the mitochondrial reference gene and the ribosomal protein L24 (*Rpl4*) as the cytoplasmic reference gene in MΦs, LPS-MΦs, and IL4-MΦ. Quantitative analysis was performed using n = 5 - 7 samples; mean values ± SEM are shown.

(D-F) BMDMΦs remained either unpolarized (MΦ) or were polarized using LPS + IFNγ (LPS-MΦ) and IL4 + IL13 (IL4-MΦ). The cells were grown for 48 hours under the following conditions: (D) physiological nutrient levels, (E) glucose shortage, or (F) glutamine shortage. The percentage of cell confluence was measured based on two-hourly scanning of the cells using the Incucyte® SX5 Live-Cell Analysis Instrument. Quantitative analysis of n = 5 shows mean values ± SEM.

(G) A representative Western blot of UCP2, actin, IkBα, and SDHA, as well as a quantification analysis of UCP2/SDHA in LPS-MΦs after 18 hours of polarization in the absence or presence of 5 µM MG132. 20 µg of total protein from each group was loaded per lane. Data are presented as mean values ± SEM.

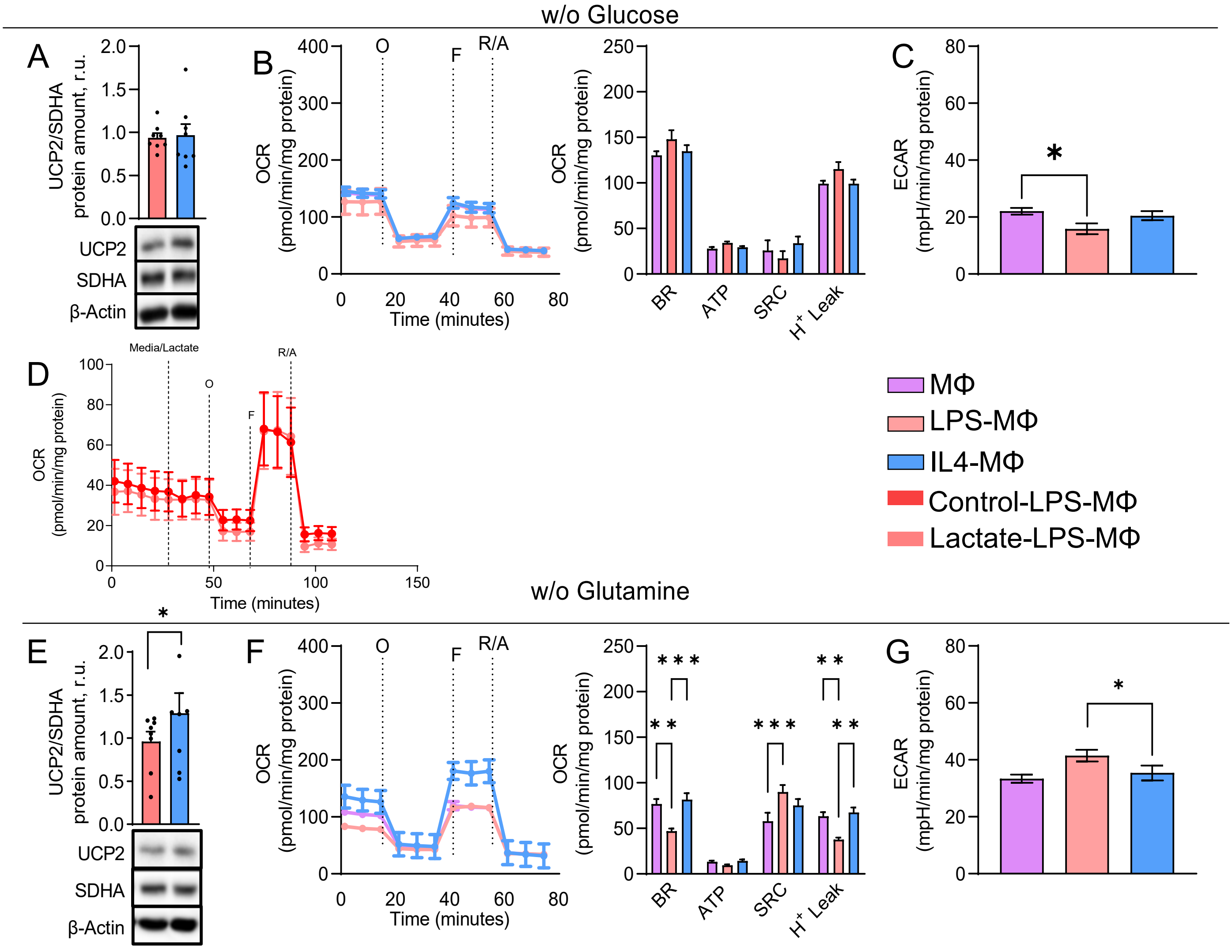

Supplementary Figure 3. Oxygen consumption rate and extracellular acidification rate of BMDMs in the absence of glucose and glutamine

(A) Quantitative analysis of UCP2 Western blot, (B) representative OCR and quantification of OCR-derived parameters and (C) ECAR in LPS-MΦs and IL4-MΦs after four hours of polarization in the absence of glucose.

(D) OCR of LPS-MΦs polarized in the absence of glucose, with or without injection of lactate.

(E) Quantitative analysis of the UCP2 Western blot, (F) representative OCR and quantification of OCR-derived parameters, and (G) ECAR in LPS-MΦs and IL4-MΦs after four hours of polarization in the absence of glucose. Data are presented as mean values ± SEM, n = 4. *$p < 0.05$, **$p < 0.01$, ***$p < 0.001$. O, oligomycin; F, FCCP; R/A, rotenone/antimycin.

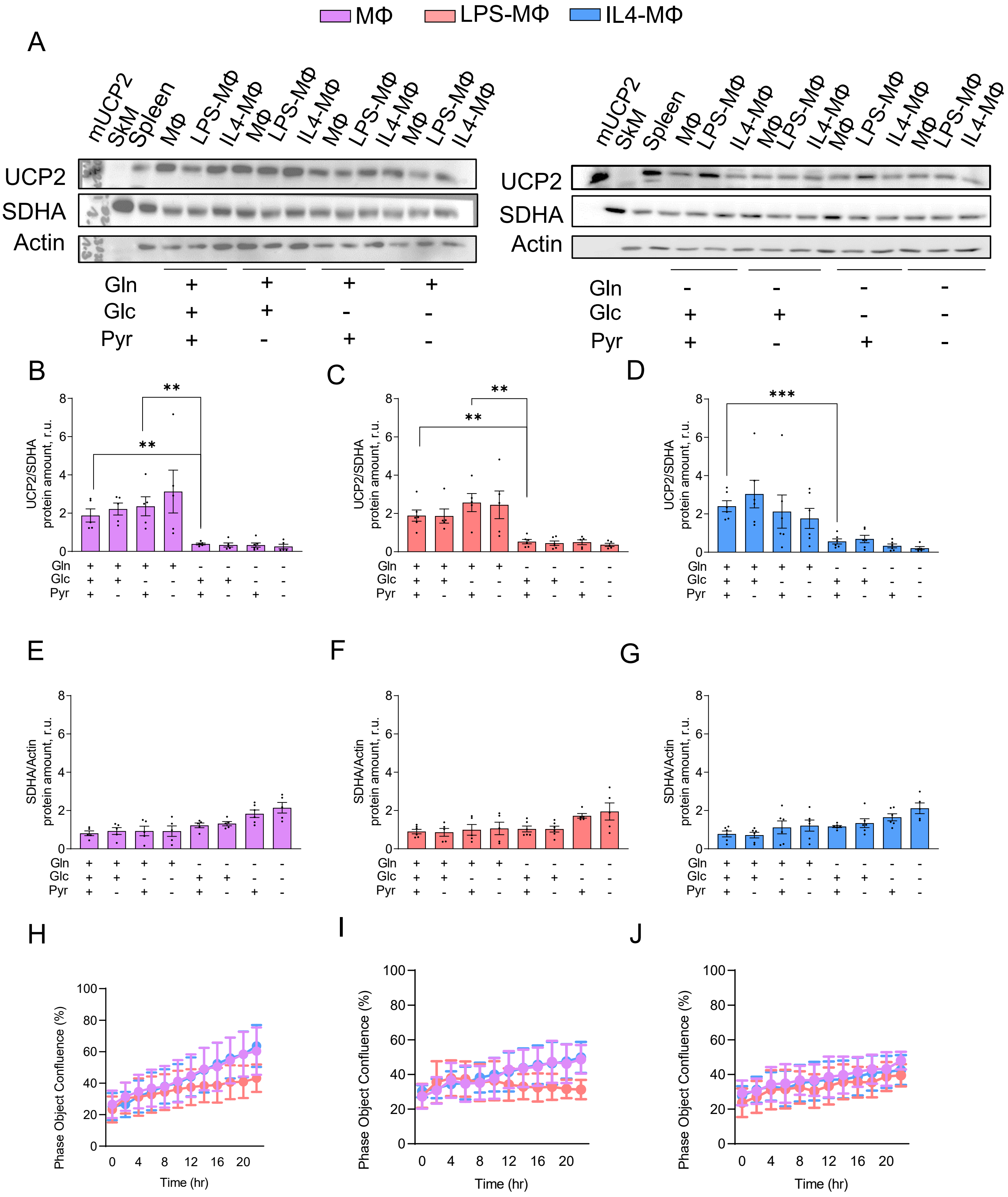

Supplementary Figure 4

Supplementary Figure 4. Correlation of the polarization state of RAW264.7 cells with UCP2 protein levels and the cell proliferation rate

The RAW 264.7 cells were either unpolarized (MΦ) or polarized using LPS/IFNɣ or IL4/IL13. The cells were then incubated for 24 hours in eight different media, some of which contained 2 mM glutamine, 5.5 mM glucose, and/or 2 mM pyruvate. (A) Representative immunoblots of total cellular protein isolated from the cells using the indicated antibodies. (B–D) Quantitative analysis of UCP2/SDHA in MΦ (B), LPS-MΦ (C), and IL4-MΦ (D). (E–G) Quantitative analysis of SDHA/actin in MΦ (E), LPS-MΦ (F), and IL4-MΦ (G). (H–J) The proliferation rate of RAW264.7 cells was evaluated for 24 hours under conditions mimicking physiological nutrient levels (H), without glucose (I), and without glutamine (J). The percentage of cell confluence was measured based on two hourly scanning of the cells using the Incucyte® SX5 Live-Cell Analysis Instrument. Quantitative analysis of n = 3 shows mean values ± SEM. Pyr, pyruvate; Glc, glucose; Gln, glutamine.

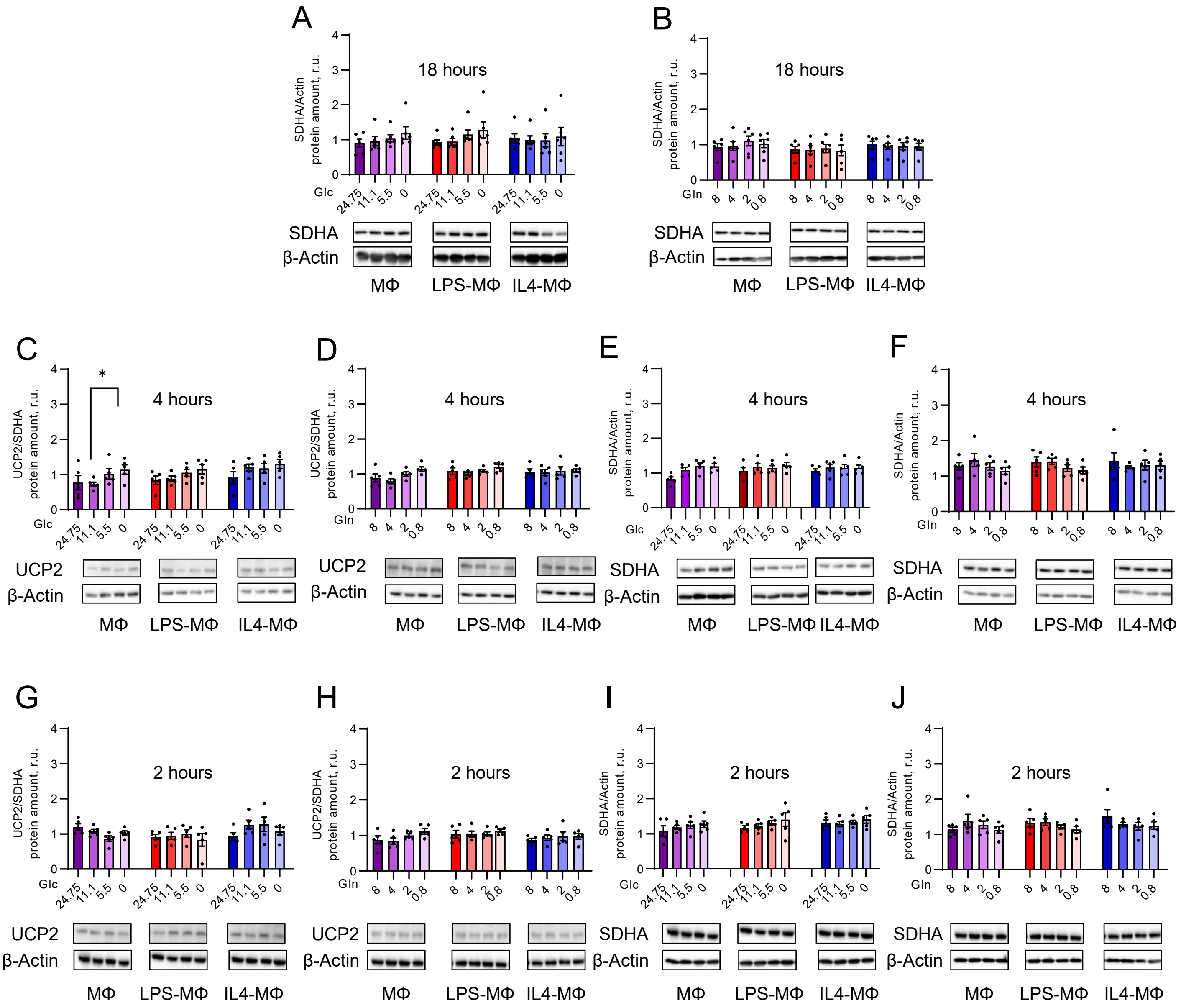

Supplementary Figure 5. Expression pattern of UCP2 and SDHA as mitochondrial control protein under different levels of glucose and glutamine.

Representative Western blots and quantification analysis of SDHA/actin in MΦs, LPS-MΦs and IL4-MΦs after (A) 18 hours (n = 6), (E) four hours, and (I) two hours (n = 5) of polarization and incubation in 24.75, 11.1, 5.5, and 0 mM of glucose and 2 mM of glutamine.

Representative Western blots and quantification analysis of SDHA/actin in nonpolarized MΦs, LPS-MΦs, and IL4-MΦs after (B) 18 hours, (F) four hours, and (J) two hours polarization in 4, 2, 0.5, and 0 mM of glutamine and 5.5 mM of glucose (n = 5).

Representative Western blots and quantification analysis of UCP2/actin in nonpolarized MΦs, LPS-MΦs, and IL4-MΦs under 24.75, 11.1, 5.5, and 0 mM of glucose and 2 mM of glutamine after (C) four hours and (G) two hours (n = 5) as well as under 4, 2, 0.5, and 0 mM of glutamine and 5.5 mM of glucose (n = 5) after (D) four hours and (H) two hours (n = 5).

20 µg of total protein isolated from each group was loaded per lane. Data are presented as mean values ± SEM.

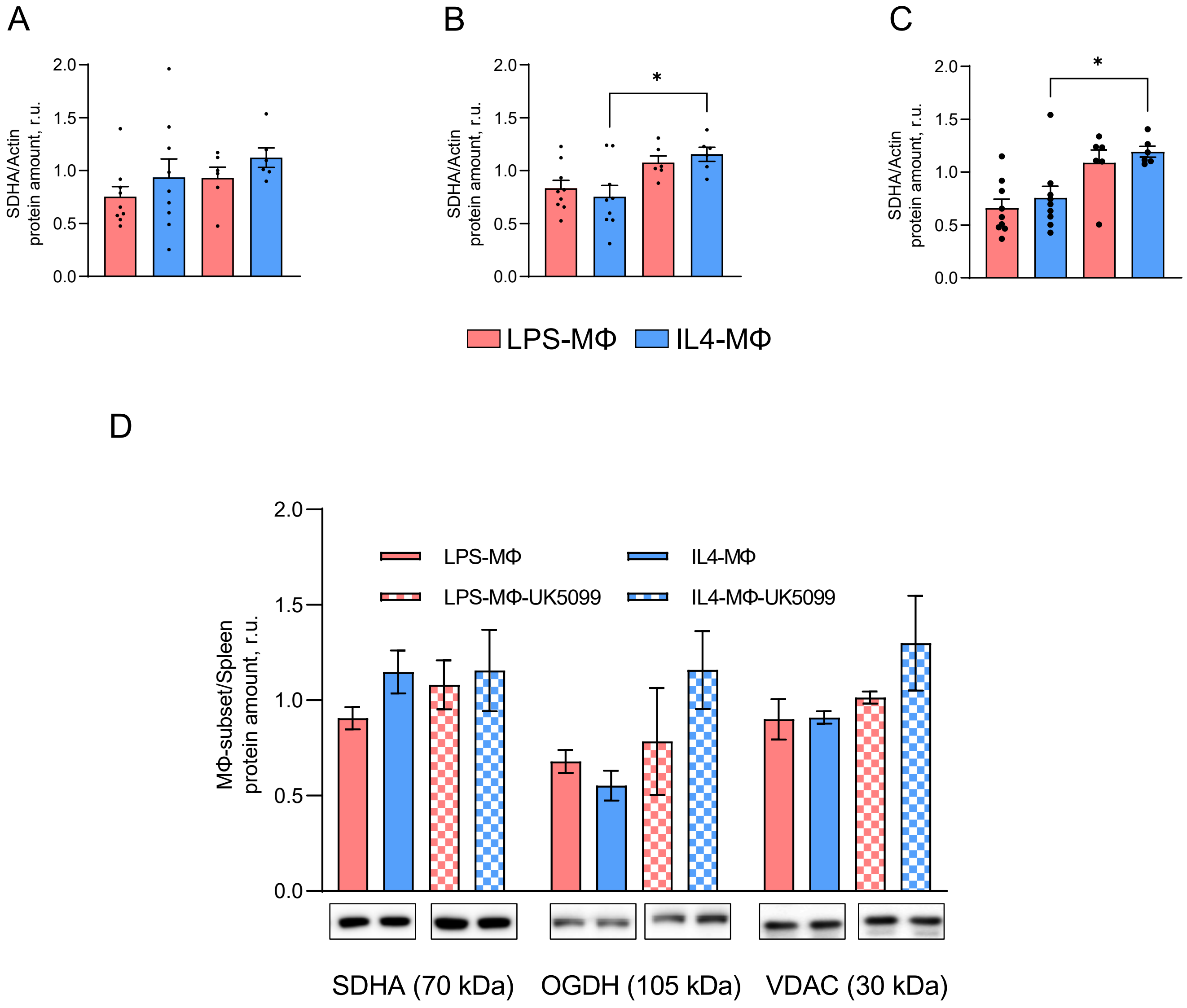

Supplementary Figure 6. **Comparison of SDHA expression as a mitochondrial control in the presence or absence of pyruvate.** Quantification analysis of SDHA/actin in LPS-MΦs and IL4-MΦs after 18 hours polarization and incubation in the following conditions: (A) physiological nutrition vs. absence of pyruvate, (B) absence of glucose vs. absence of glucose and pyruvate, and (C) absence of glutamine vs. absence of glutamine and pyruvate (n = 6 - 9). (D) Representative Western blots and quantification analysis of SDHA in LPS-MΦs and IL4-MΦs relative to spleen after 18 hours polarization and incubation in the absence or presence of 5 μM UK-5099. 20 μg of isolated total protein from each group was loaded per lane. Data are presented as mean values ± SEM, *$p < 0.05$.

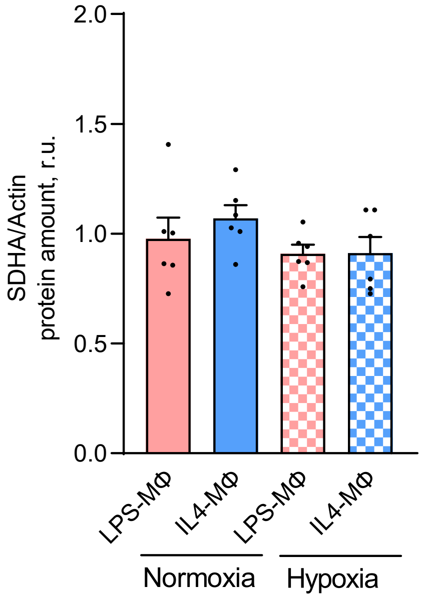

Supplementary Figure 7. Evaluation of SDHA levels under hypoxia.

Quantification analysis of SDHA/actin in LPS-MΦs and IL4-MΦs after 18 hours polarization and incubation in the physiological nutrition under normoxic versus hypoxic conditions (n = 7). 20 µg of isolated total protein from each group was loaded per lane.

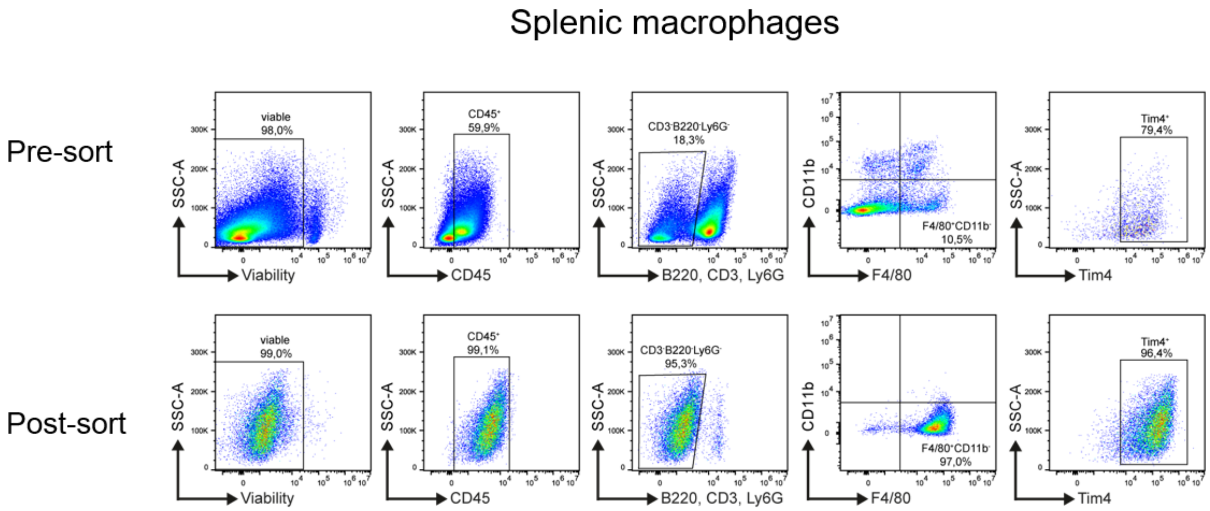

Supplementary Figure 8. Flow cytometry gating strategy and purity assessment of splenic macrophages before and after magnetic enrichment. Representative flow cytometry plots showing the sequential gating steps used to identify and enrich splenic macrophages. Pre-sort: Total splenocyte suspension prior to enrichment, with gates for viable cells, $CD45^+$ leukocytes, exclusion of $CD3^+$/$B220^+$/$Ly6G^+$ non-myeloid cells, and identification of $F4/80^+CD11b^+$ macrophages, followed by Tim4 expression. Post-sort: Cell population after the two-step APC/PE magnetic enrichment, demonstrating a marked increase in purity, with >95% of cells expressing F4/80, CD11b, and Tim4. This gating confirms the effectiveness of the bead-based strategy in isolating highly enriched tissue-resident macrophages from the spleen.

Supplementary Table 1. Antibodies/markers used for the two-step magnetic enrichment of the respective TRM population.

TRM	Antibody/Marker
Splenic macrophages	F4/80-APC, Tim4-PE
Monocytes	CD11b-APC, CD115-PE
Peritoneal macrophages	Tim4-PE, F4/80-APC
Kupfer cells	F4/80-APC, Tim4-PE
Alveolar macrophages	CD11c-PE, SiglecF-APC
Adipose Tissue macrophages	F4/80-APC, Tim4-PE
Microglia	Myelin removal, P2RY12-PE
Colonic macrophages	CX3CR1-APC, MHC-II-PE

Supplementary Table 2. Primer sequences used for qRT-PCR.

Gene	Primer Sequence
Ucp2	F: 5'-AAAGCAGCCTCCAGAACTCCG-3'
	R: 5'-TTCACAGTGGCTGTTGGGGG-3'
Rpl4	F: 5'-GTATGGCACTTGGCGGAAGG-3'
	R: 5'-TGCTCGGAGGGCTCTTTGG-3'
mRpl24	F: 5'-TGAGCCGTCCAGGTTCCATA-3'
	R: 5'-ACAGAATAGGTGCCAGTCTTCA-3'
Hif1a	F: 5'-AGG ATG AGT TCT GAA CGT CGA AAA G-3'
	R: 5'-CAC TGT CTA GAC CAC CGG CA-3'
Egln1	F: 5'-AAT TCG GCA CGA GGG CAA GT-3’
	R:5'-CAG TGG CGG ATC AGG TCG TC-3'
