# Supplementary material for: Pro‐ and Anti‐Inflammatory Macrophages Adjust UCP2 Protein Levels Based on Their Intrinsic Metabolism and Available Metabolites
Source: Eur J Immunol. 2026 Jun 9;56(6):e70218. doi: 10.1002/eji.70218 (PMC13247729; doi:10.1002/eji.70218)

Original Western Blots

Fig 1B. Original western blot

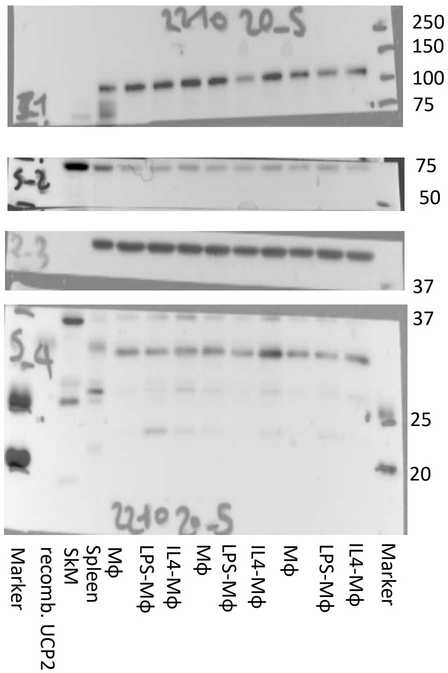

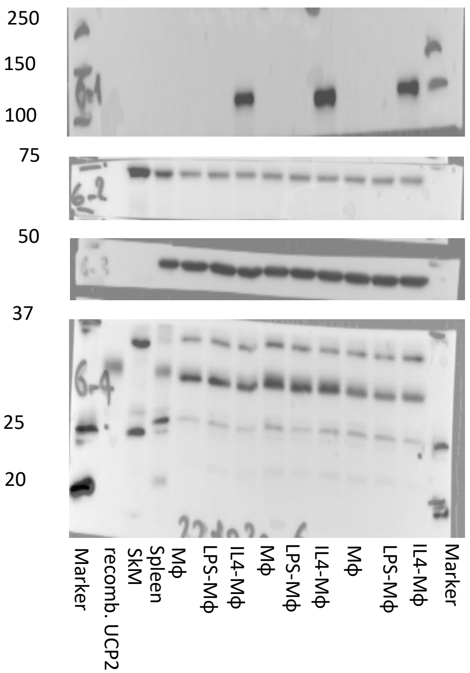

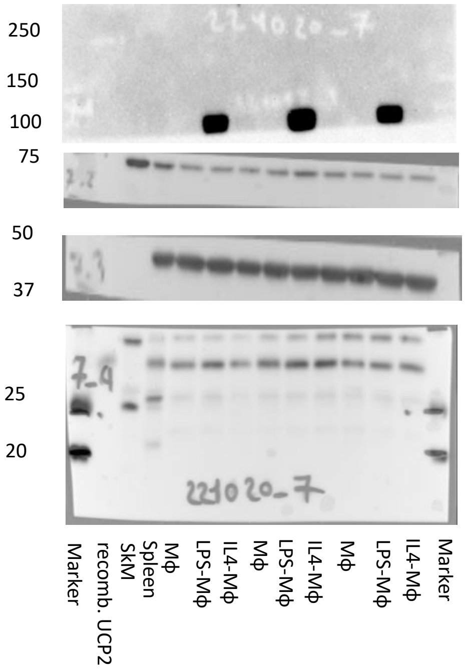

Fig 1C. Original western blot

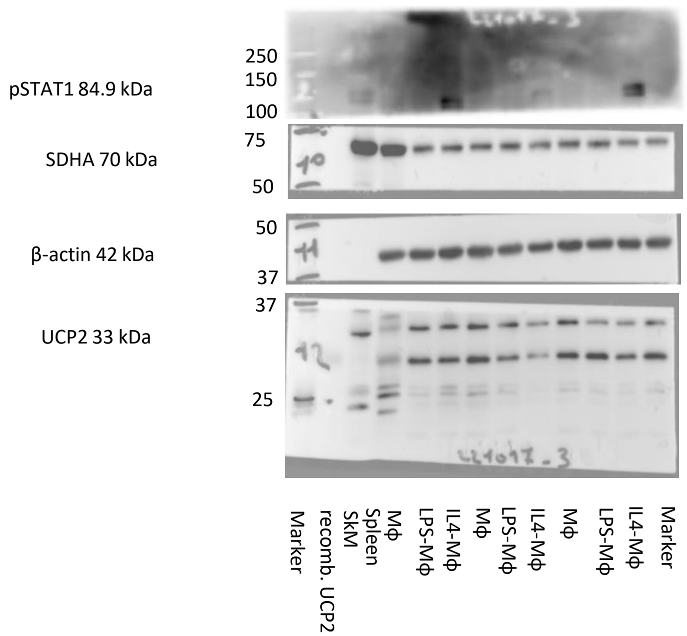

Fig 2E. Original western blot

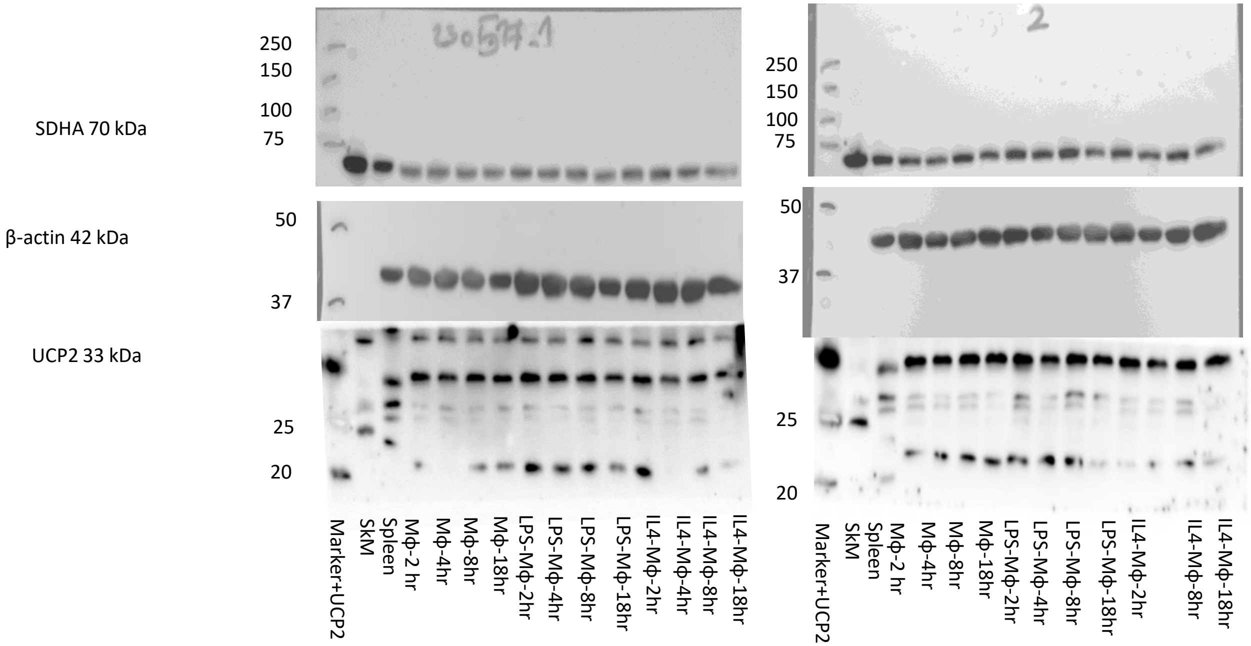

Fig 2A, 3A, and 3E. Original western blot

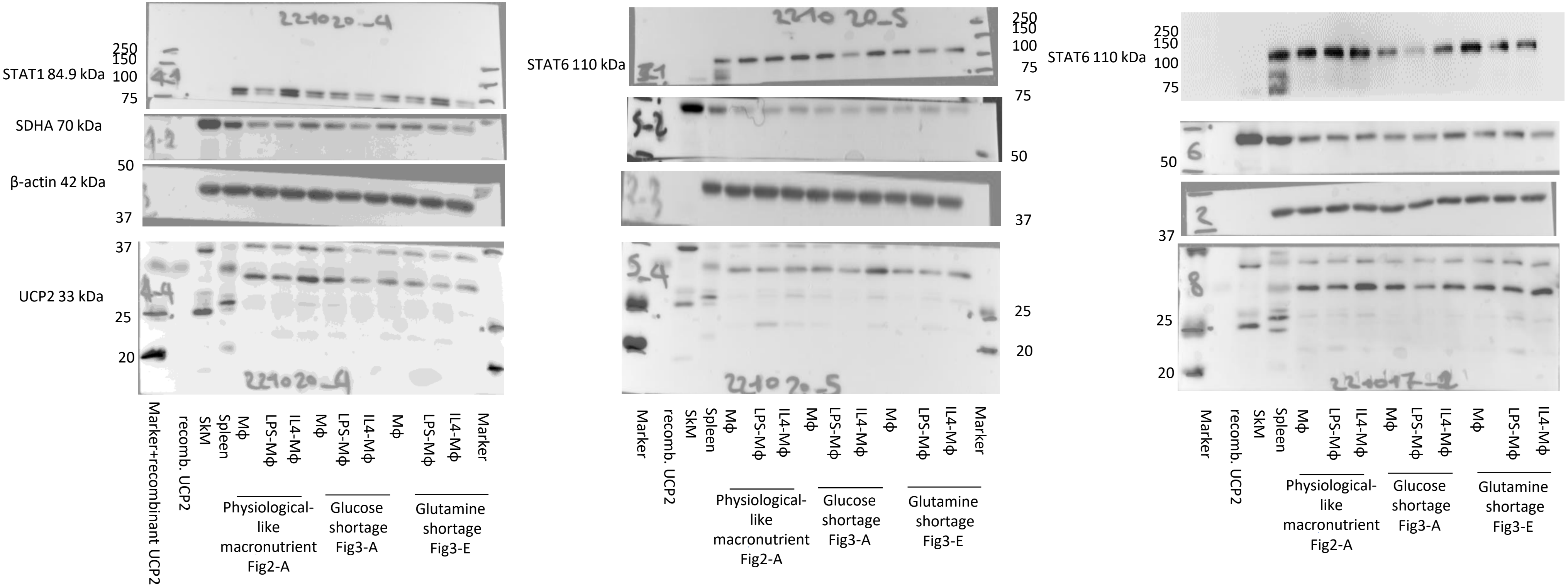

Fig 2E and 3A and E. Original western blot

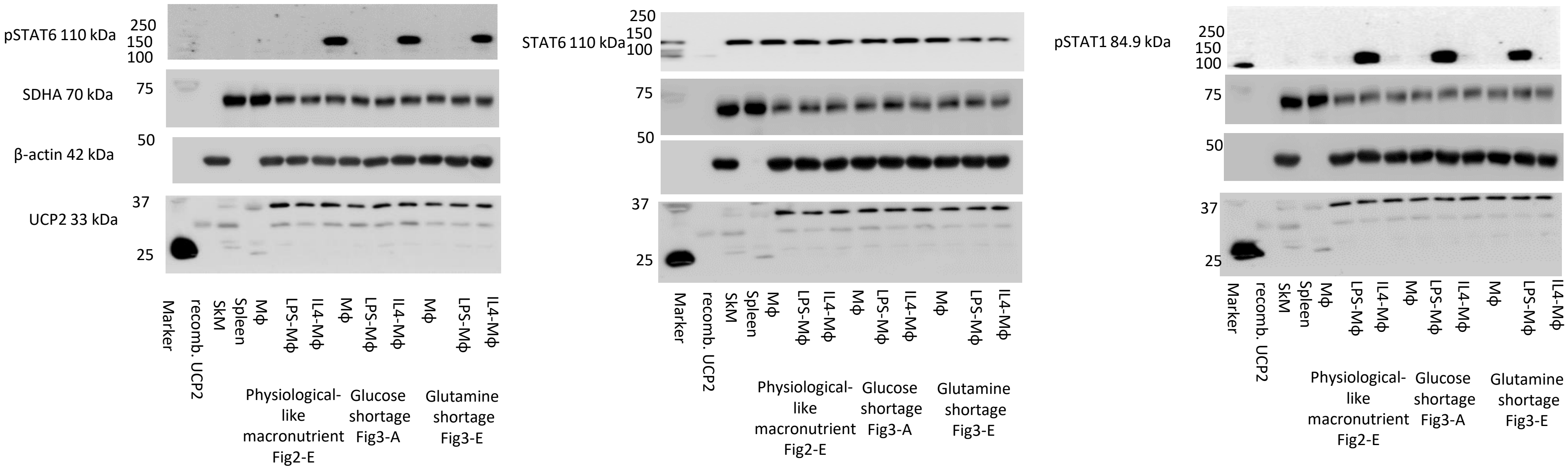

Fig 4A. Original western blot

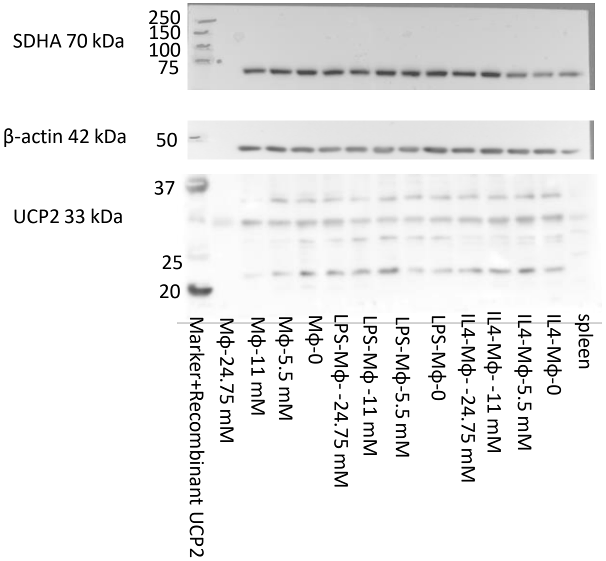

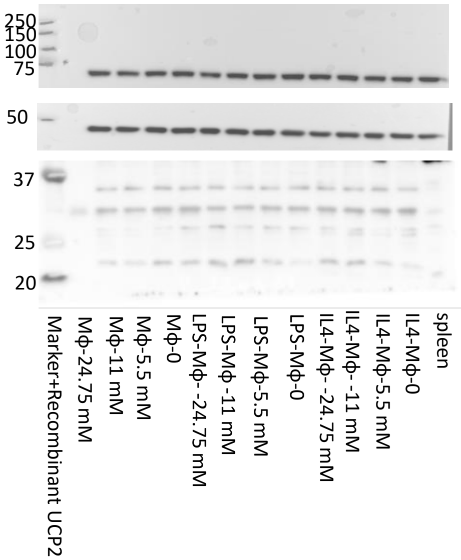

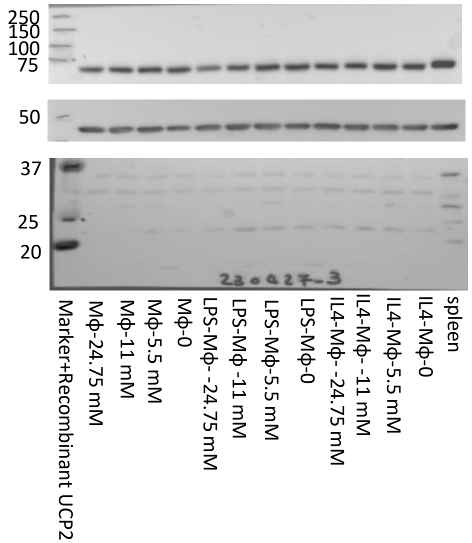

Fig 4B. Original western blot

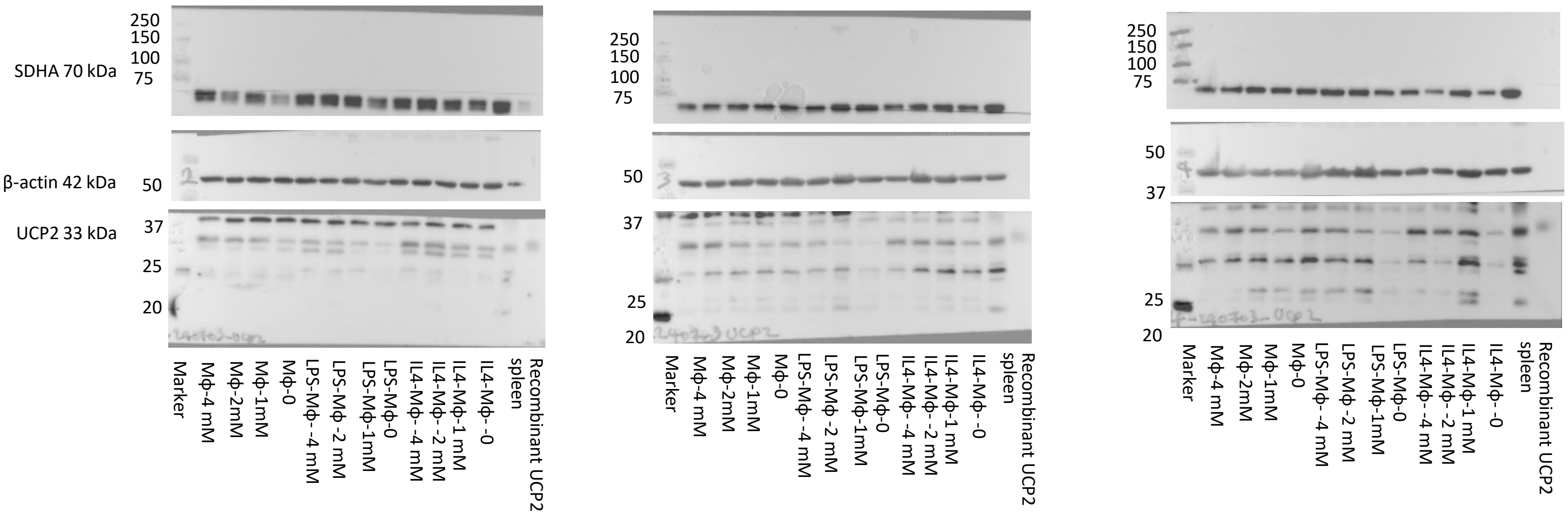

Fig 5 A, B, and C. Original western blot

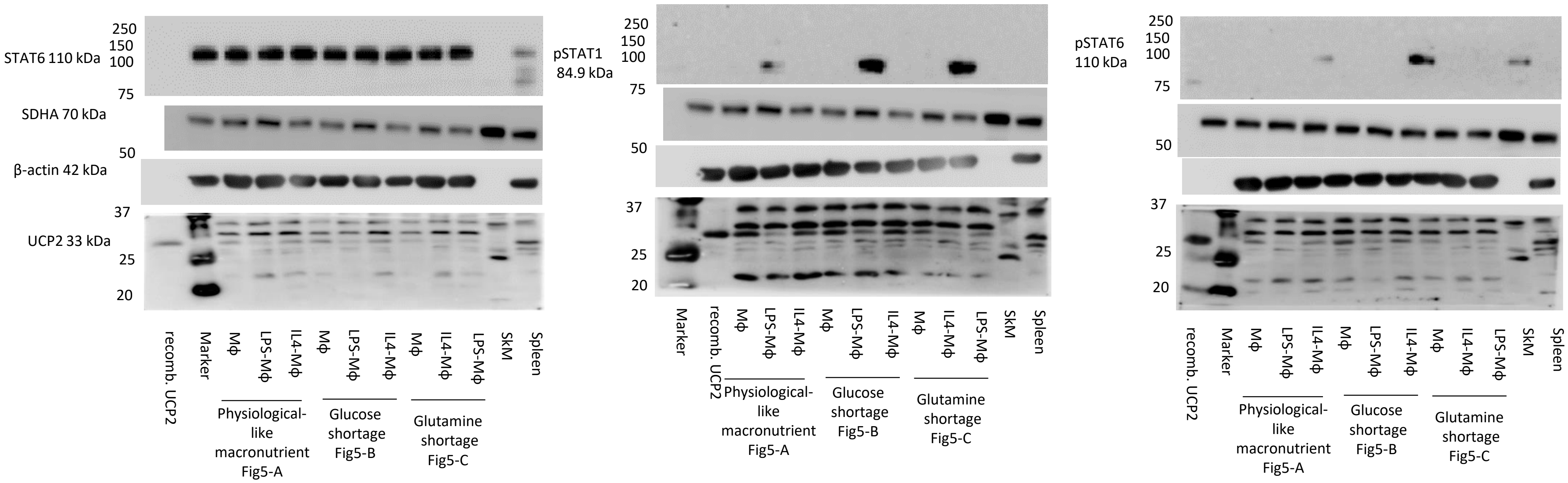

Fig 5 D. Original western blot

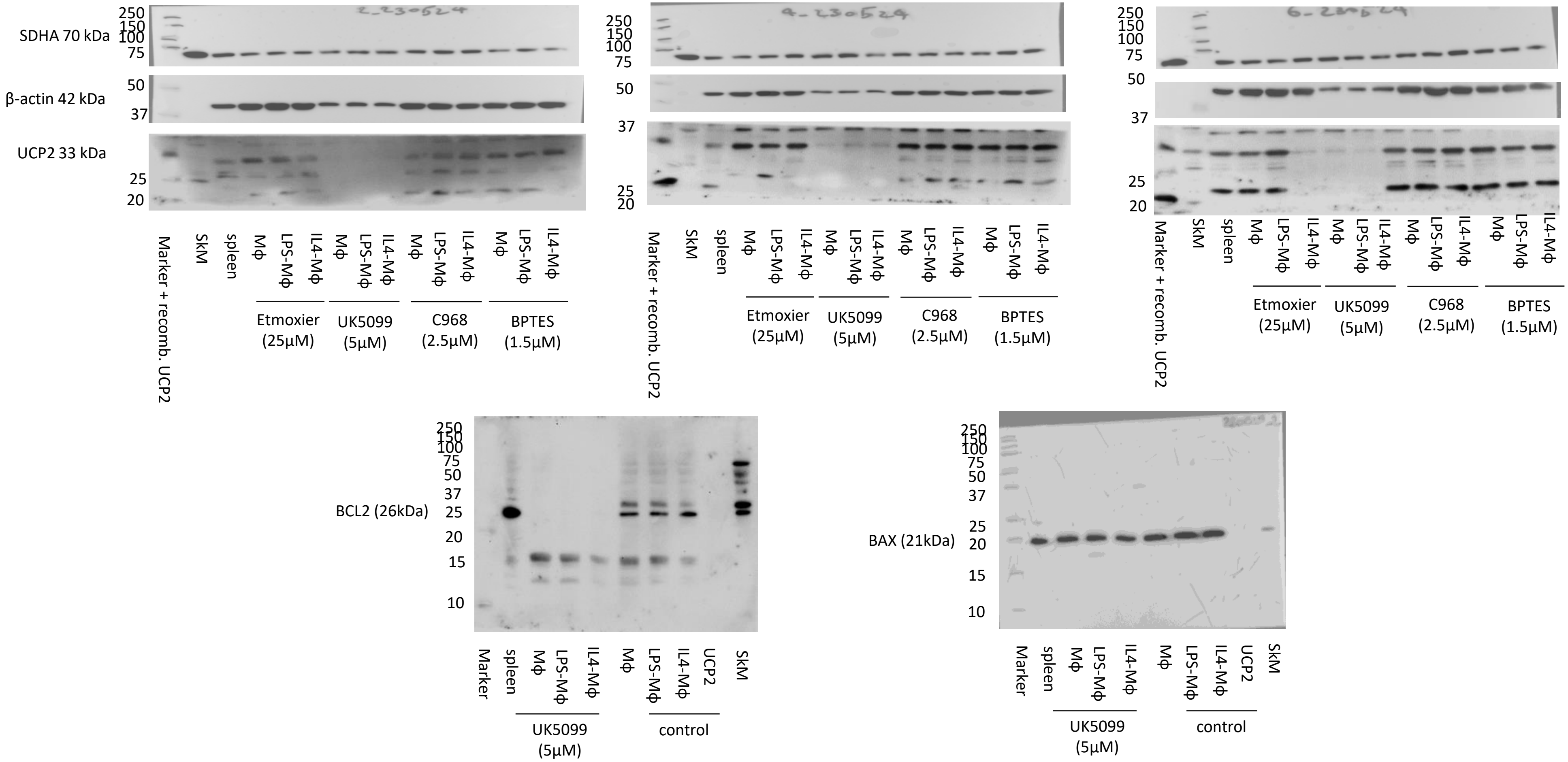

Fig 5 E. Original western blot

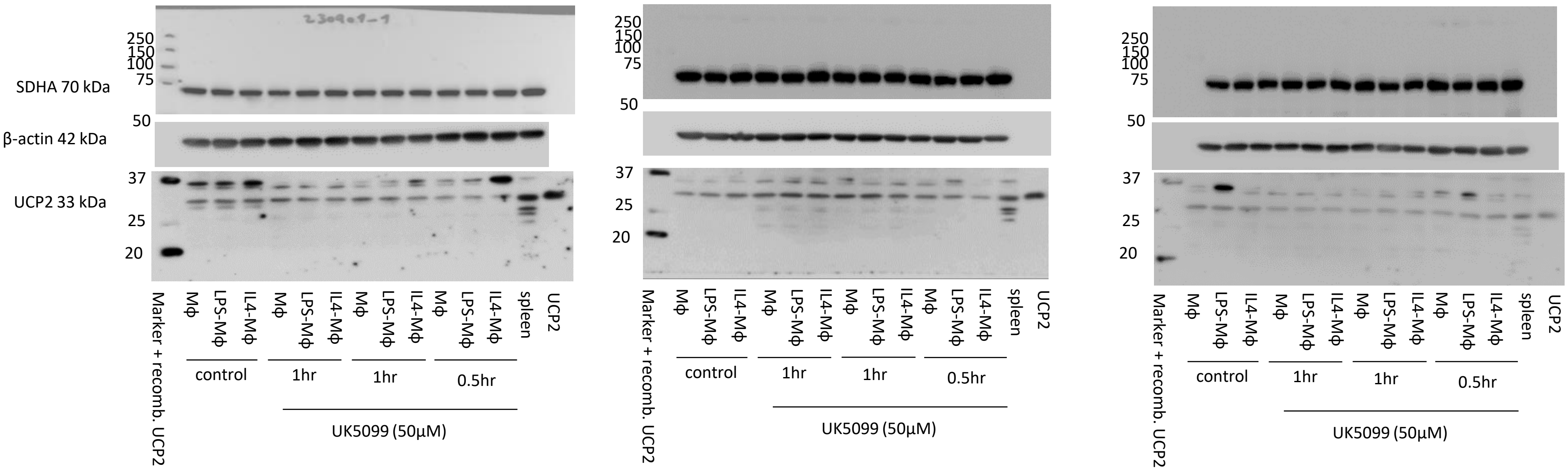

Fig 6A. Original western blot

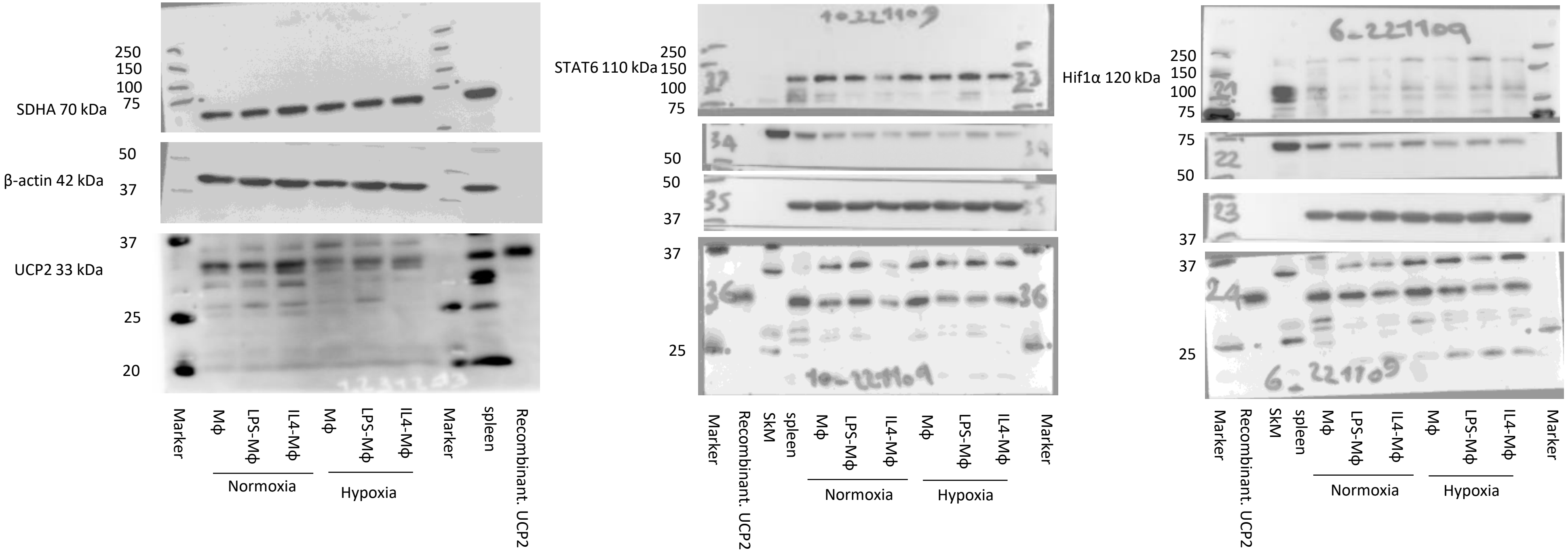

Supplement: Supplementary file 2 — Supporting File 2: eji70218‐sup‐0002‐SuppMat.pdf. [file EJI-56-e70218-s002.pdf]
